# A lincRNA-p21/miR-181 family feedback loop regulates microglial activation during systemic LPS- and MPTP- induced neuroinflammation

**DOI:** 10.1038/s41419-018-0821-5

**Published:** 2018-07-23

**Authors:** Yongyi Ye, Xiaozheng He, Fengfei Lu, Hengxu Mao, Zhiyuan Zhu, Longping Yao, Wanxian Luo, Xiang Sun, Baoyan Wang, Chen Qian, Yizhou Zhang, Guohui Lu, Shizhong Zhang

**Affiliations:** 1 The National Key Clinical Specialty, The Engineering Technology Research Center of Education Ministry of China, Guangdong Provincial Key Laboratory on Brain Function Repair and Regeneration, Department of Neurosurgery, Zhujiang Hospital, Southern Medical University, 510282 Guangzhou, China; 2 Department of Medicine Ultrasonics, Nanfang Hospital, Southern Medical University, 510515 Guangzhou, China; 3 Tarbut v’torah community day school, Irvine, CA 92603 USA; 4 Department of Neurosurgery, the First Affiliated Hospital of Nanchang University, 330006 Nanchang, China

## Abstract

The role of microglial-mediated sustained neuroinflammation in the onset and progression of Parkinson’s disease (PD) is well established, but the mechanisms contributing to microglial activation remain unclear. LincRNA-p21, a well studied long intergenic noncoding RNA (lincRNA), plays pivotal roles in diverse biological processes and diseases. Its role in microglial activation and inflammation-induced neurotoxicity, however, has not yet been fully elucidated. Here, we report that lincRNA-p21 promotes microglial activation through a p53-dependent transcriptional pathway. We further demonstrate that lincRNA-p21 competitively binds to the miR-181 family and induces microglial activation through the miR-181/PKC-δ pathway. Moreover, PKC-δ induction further increases the expression of p53/lincRNA-p21 and thus forms a circuit. Taken together, our results suggest that p53/lincRNA-p21, together with miR-181/PKC-δ, form a double-negative feedback loop that facilitates sustained microglial activation and the deterioration of neurodegeneration.

## Introduction

Parkinson’s disease (PD) is the second most common neurodegenerative disorder, with evolving layers of complexity. The underlying molecular mechanisms of PD are still poorly understood. In recent years, neuroinflammation, essentially mediated by chronic and sustained activated microglia, has been receiving particular attention^1–3^. Microglia is believed to exacerbate the loss of dopaminergic neurons within the SN once they are persistently activated^4,5^. Thus, understanding the regulation of microglial activation is critical for comprehending the extant inflammatory response during the onset and progression PD.

LncRNAs are considered to serve as a cryptic, but critical layer in the genetic regulatory code^6^ and are associated with diverse physiological and pathological responses^7^. Significant insight has been gained regarding their potential importance in the immune system^8,9^. For example, in a recent report, lncRNA GAS5 has been reported to suppress microglial M2 polarization by inhibiting the transcription of TRF4, a key factor controlling M2 macrophage polarization^10^. Although numerous lncRNAs are induced in innate immune cells, many of them remains functionally uncharacterized.

Four archetypes of molecular mechanisms have been proposed to illustrate the myriad functions of lncRNAs-signals, decoys, guides, and scaffolds, in which lncRNAs interact with DNA or RNA through nucleic-acid base pairing or with proteins through modular domains^11^. Specifically, a new hypothesis, namely competing endogenous RNAs (ceRNAs), has been proposed for the layer of gene regulation mediated by RNA transcripts with shared miRNA binding sites (MREs)^12^. Based on this hypothesis, lncRNAs are believed to fine tune gene expression through this new “RNA language”. Increasing evidence indicates that disruption of the equilibrium of ceRNA networks can be critical for various diseases and developmental stages^13^. As one of the ceRNA protagonists, microRNAs (miRNAs) are also well known key modifiers for fine tuning key genetic pathways in microglial polarization and function^14,15^. Therefore, we propose that some lncRNAs may interact with miRNAs and act as ceRNAs in the post-transcriptional network in microglia.

LincRNA-p21 was previously identified as a p53-inducible lncRNA and functions as a component of p53-dependent transcriptional responses^16^. However, the functions and mechanisms utilized by lincRNA-p21 during microglial activation and the potential impact of lincRNA-p21 on the inflammatory component of PD have not as yet been fully elucidated. In this study, we thus focused on the role of the lincRNA-p21-mediated ceRNA network in microglial activation and inflammatory responses in PD.

## Results

### LincRNA-p21 promotes microglial activation in vitro

**Fig. 1:**
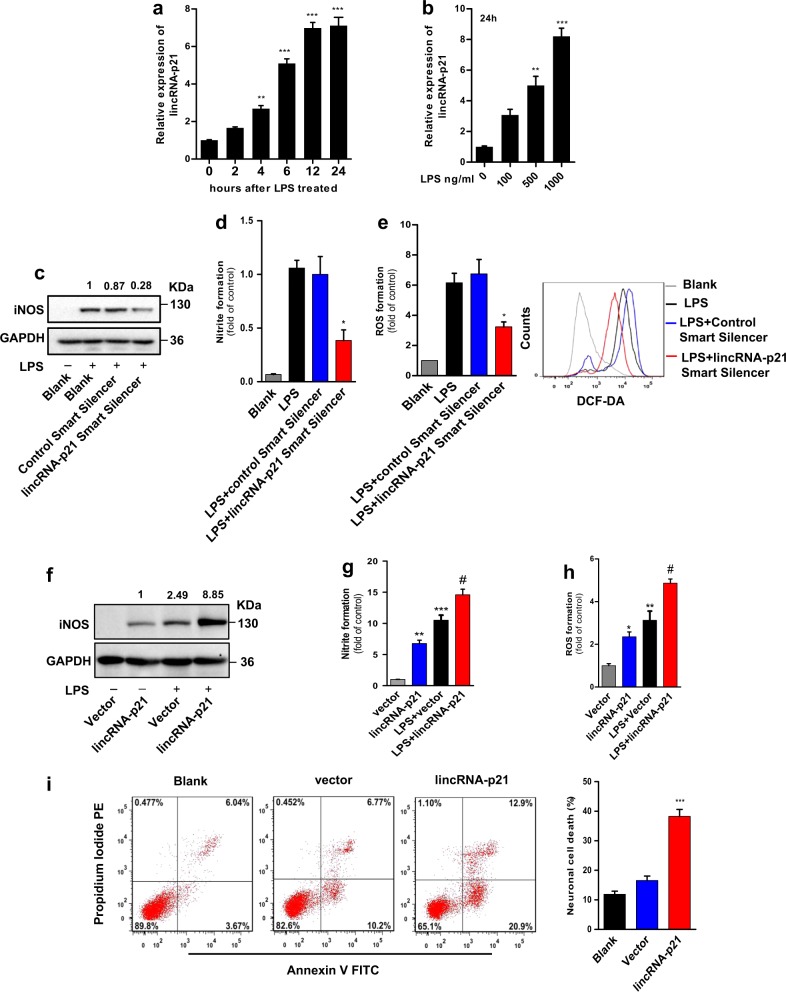
LincRNA-p21 regulates microglial activation in vitro. **a**, **b** BV2 microglia cells were treated with LPS and expression levels of lincRNA-p21 were analysed by qRT-PCR. LincRNA-p21 levels in response to LPS treatment are time-(**a**) and dose-dependent (**b**) in BV2 microglia cells. **c–e** Effect of lincRNA-p21 knockdown on LPS-induced microglial activation. BV2 microglia cells were transfected with lincRNA-p21 Smart Silencer or control Smart Silencer and exposed to LPS for 12 h. Cells were harvested and assessed for iNOS expression (**c**), NO production (**d**) and ROS formation (**e**). **f–h** Effect of lincRNA-p21 overexpression on microglial activation. BV2 microglia cells were transfected with lincRNA-p21-overexpressing vector or control vector and exposed to LPS. Cells were harvested and assessed for iNOS expression (6 h) (**f**), NO production (24 h) (**g**) and ROS formation (6 h) (**h**). **i** Conditioned medias (CMs) from lincRNA-p21 overexpression group induces more SH-SY5Y cell death. Representative images of SH-SY5Y cell death by flow cytometry (left panel) and quantification of SH-SY5Y cell death (right panel). Data are expressed as mean ± SEM (*n* = 3). *P < 0.05, **P < 0.01, ***P < 0.001 versus control; ^#^*P* < 0.05 versus control group treated with LPS using one-way analysis of variance ANOVA followed by Bonferroni test

We began by assessing lincRNA-p21 levels in LPS-treated BV2 microglia cells. LPS treatment induced lincRNA-p21 levels in a time-and dose-dependent manner (Fig. 1a, b). Increased lincRNA-p21 expression also observe upon treatment with other pro-inflammatory stimulus such as lipoteichoic acid (LTA, TLR2 agonist), PamC3sk4 (synthetic lipopeptide TLR1/2 agonist), and interferon-gamma (IFN-γ) (Supplementary Fig. 1k). We then transfected BV2 microglia cells with lincRNA-p21 Smart Silencer to knock down its endogenous expression and testify the effect of lincRNA-p21 on microglial activation. As shown in Fig. 1c–e, Supplementary Fig. 1a, d, LPS-treated lincRNA-p21-depleted BV2 microglia cells displayed decreased inducible nitric oxide synthases (iNOS) expression, NO production, reactive oxygen species (ROS) formation and cytokines induction (IL-6, TNFa, IL-1β, and MCP-1). Similar results were also observed with LTA, PamC3sk4 and IFN-γ treatments (Supplementary Fig. 1l‒o). In addition, to demonstrate that the observed derepression upon lincRNA-p21 knockdown was not due to off-target effects of the RNAi-mediated knockdown, we repeated the knockdown experiments with another two small interfering RNAs (siRNAs) targeting lincRNA-p21 and obtained similar results (Supplementary Fig. 1b, e‒j). Moreover, as shown in Fig. 1f–h and Supplementary Fig. 1c, transient overexpression lincRNA-p21 induced iNOS, NO and ROS formation, further potentiated LPS-mediated microglial activation in BV2 microglia cells. Finally, a conditioned medium (CM) transfer system was used to determine the influence of lincRNA-p21 on microglial-mediated neurotoxicity. As shown in Fig. 1i, CM from lincRNA-p21 overexpression group resulted in more SH-SY5Y cells death. Collectively, these results indicate that lincRNA-p21 is critical for the activation of microglia.

### The effect of lincRNA-p21-induced microglial activation is p53-dependent

**Fig. 2:**
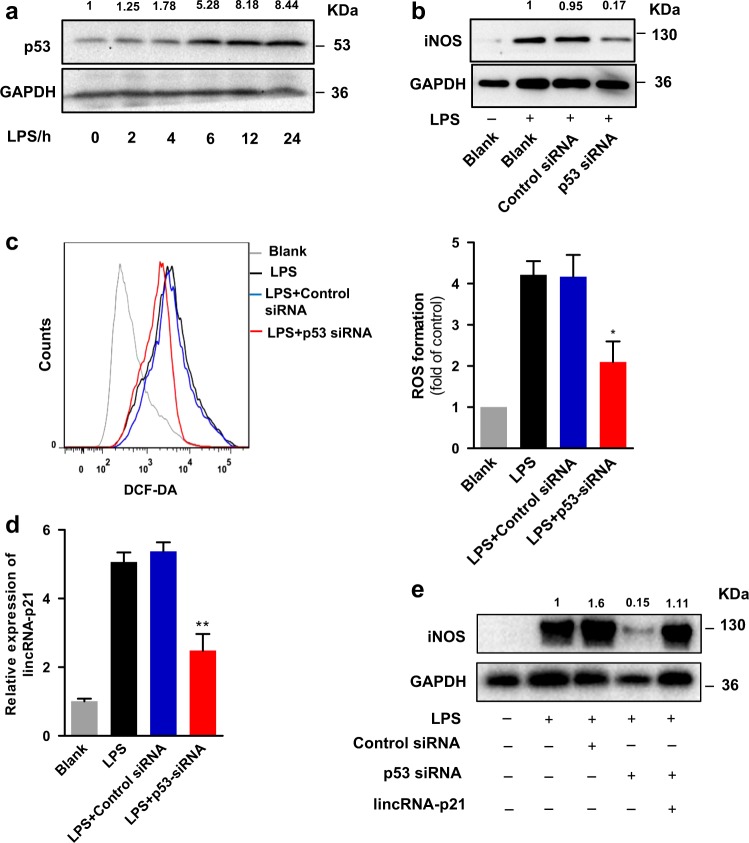
LincRNA-p21 promotes microglial activation in a p53-dependent pathway. **a** Western blotting analysis of p53 expression in BV2 microglia cells treated with LPS for different time points. **b**, **c** Effect of p53 knockdown on LPS-induced microglial activation. BV2 microglia cells were transfected with p53 specific siRNA or control siRNA and exposed to LPS for 12 h. Cells were harvested and assessed for iNOS expression (**b**), and ROS formation (**c**). **d** qRT-PCR analysis of lincRNA-p21 in BV2 microglia cells treated with LPS following transfection of cells with p53 specific siRNA or control siRNA. **e** BV2 microglia cells were co-transfected with p53 specific siRNA or control siRNA and lincRNA-p21-overexpressing vector and exposed to LPS. Cells were then harvested and assessed for iNOS expression by western blotting. Data are expressed as mean ± SEM (*n* = 3). *P < 0.05, **P < 0.01 versus control using one-way analysis of variance ANOVA followed by Bonferroni test

Earlier studies^17,18^ have demonstrated that p53 mediates microglial activation and triggers microglial-induced neurotoxicity in many neurodegenerative diseases. Indeed, we found that exposure of BV2 microglia cultures to LPS led to significantly increased p53 protein expression (Fig. 2a). Knockdown of p53 using siRNA resulted in a reduction of iNOS expression and ROS formation upon LPS treatment (Fig. 2b, c, Supplementary Fig. 2a). Therefore, we sought to testify the observed lincRNA-p21-mediated microglial activation was indeed p53 dependent. As expected, knockdown of p53 significantly impaired the LPS-induced lincRNA-p21 transcripts in BV2 microglia cells (Fig. 2d). Ectopic expression of lincRNA-p21 prevented the reduction of iNOS resulting from p53 knockdown (Fig. 2e), and vice versa, silencing of p53 and lincRNA-p21 simultaneously reduced iNOS expression even further (Supplementary Fig. 2b). Taken together, these data suggest that the effect of lincRNA-p21 on microglial activation is, at least in part, dependent on the p53 pathway.

### LincRNA-p21 physically associates with the miR-181 family

**Fig. 3:**
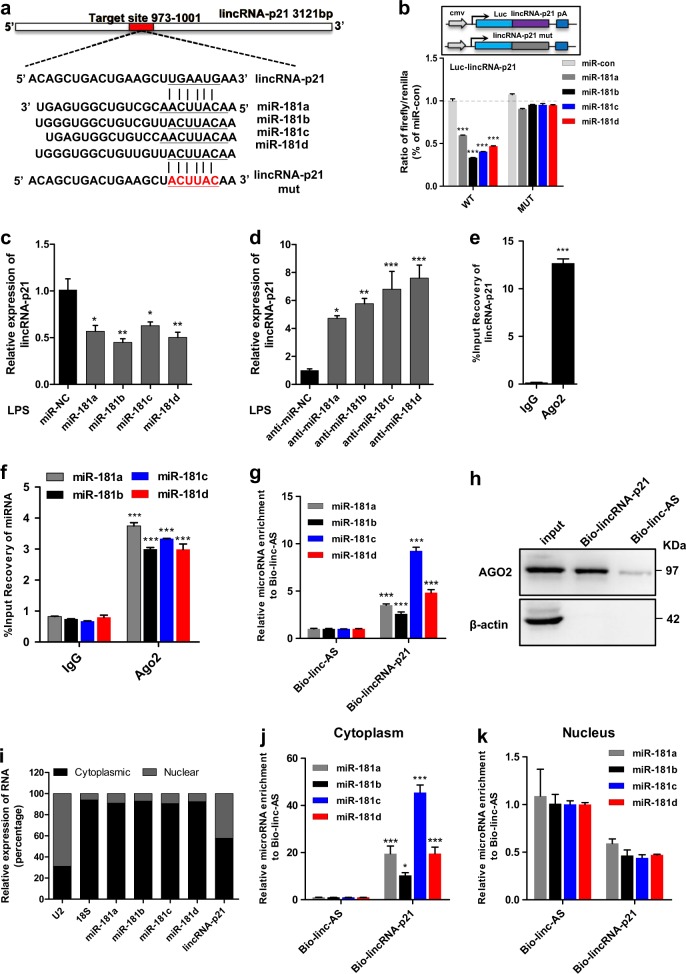
LincRNA-p21 physically associates with the miR-181 family. **a** Putative miR-181 family binding site in lincRNA-p21. The potential complementary residues are underlined and mutated bases are indicated in red. **b** Upper panel, schematic representation of pmirGLO-based luciferase reporter plasmid containing wild-type lincRNA-p21 (Luc-lincRNA-p21-wt) and a mutant reporter construct (Luc-lincRNA-p21-mut). Lower panel, relative luciferase activity of the luciferase constructs of Luc-lincRNA-p21-wt or Luc-lincRNA-p21-mut co-transfected with miR-control/miR-181 family in HEK293 cells. A representative experiment with triplicates of two independent experiments is shown. Data are expressed as mean ± SEM of triplicate wells. *P < 0.05, **P < 0.01 in one-way analysis of variance ANOVA followed by Bonferroni test. **c**, **d** qRT-PCR analysis of lincRNA-p21 in BV2 microglia cells treated with LPS following transfection of cells with miR-control/miR-181a, b, c, d mimics (**c**); or anti-miR-control/anti-miR-181 a, b, c, d (inhibitors) (**d**). Data are expressed as mean ± SEM (*n* = 3). *P < 0.05, **P < 0.01, ***P < 0.001 versus control using one-way analysis of variance ANOVA followed by Bonferroni test. **e**, **f** Anti-Ago2 RIP was performed in BV2 microglia cell lysates. Co-precipitated RNAs were used to detect the relative enrichment of lincRNA-p21 (**e**) and miR-181 family (**f**) associated with Ago2, compared with that in normal IgG control. **g**, **h** In vitro-synthesized biotin-labeled RNA pulldown experiments in BV2 microglia cells. BV2 microglia cell lysates were incubated with in vitro-synthesized biotin-labeled lincRNA-p21 sense or antisense RNA for biotin pull-down assay, followed by qRT-PCR to detect miR-181 family (**g**) and western blotting to detect Ago2 (**h**) associated with lincRNA-p21. Bio-linc-AS, biotinylated-lincRNA-p21-antisense RNA; bio-lincRNA-p21, biotinylated-lincRNA-p21-sense RNA. **i** Representative analysis of lincRNA-p21 and miR-181 family distribution in nuclear and cytoplasmic fractions in BV2 microglia cells. U2 snRNA and 18S rRNA served as controls for nuclear and cytoplasmic RNA, respectively. Exogenous spike-in of cel-mir-39 served as a normalizer for the qRT-PCR analysis. **j**, **k** Biotin pull-down assay with biotin-labeled lincRNA-p21 sense or antisense RNA was performed with the cellular fractions of cytoplasm (**j**) or nucleus (**k**) of BV2 microglia cells. A representative experiment with triplicates of two independent experiments is shown. Data are expressed as mean ± SEM of triplicate wells. *P < 0.05, **P < 0.01 ***P < 0.001 in two-way analysis of variance ANOVA followed by Bonferroni test

The recent ceRNA hypothesis proposes that lincRNAs can function as s miRNA sponges, functionally liberating other transcripts with MREs^19^. Bioinformatics prediction using Segal lab (http://132.77.150.113/pubs/mir07/mir07_prediction.html) suggested that lincRNA-p21 presented a putative binding site for the miR-181 family (miR-181a/b/c/d) (Fig. 3a). Importantly, all the miR-181 family members have been reported to functionally regulate the inflammatory phenotype^20–23^. Therefore, we performed dual-luciferase assays and found that all the miR-181 family members greatly reduced the luciferase activities of Luc-lincRNA-p21-wt but not Luc-lincRNA-p21-mut (Fig. 3b). We also observed a significant decrease in lincRNA-p21 levels after overexpression of miR-181 family (Fig. 3c), while a significant increase after knockdown of miR-181 family (Fig. 3d) in response to LPS. These results reveal that miR-181 family may interact with lincRNA-p21 and induce the degradation of lincRNA-p21.

To further validate the direct binding between miR-181 family and lincRNA-p21 at endogenous levels, we performed an anti-Ago2 RNA immunoprecipitation (RIP) in BV2 microglia cells extracts. As expected, we found that both miR-181a/b/c/d and lincRNA-p21 were specifically enriched in the Ago2 group (Fig. 3e, f). Moreover, we also performed a biotin–Avidin pull-down assay to further validate the specific association between miR-181 family and lincRNA-p21. Using in vitro-transcribed biotin-labeled lincRNA-p21, we found that endogenous miR-181a/b/c/d was significantly pulled down (Fig. 3g). Ago2 enrichment was also observed in this lincRNA-p21-pull-down complex (Fig. 3h), indicating that lincRNA-p21 is recruited to Ago2-related RNA-induced silencing complexes and functionally interacts with miR-181 family. We further found that lincRNA-p21 was expressed both in the nucleus and cytoplasm, while miR-181a/b/c/d and Ago2 were mainly expressed in the cytoplasm (Fig. 3i; Supplementary Fig. 3a–d). Hence, to further determine whether the interaction between lincRNA-p21 and miR-181 family occurred in the cytoplasm, a biotin–Avidin pull-down assay was performed in the nuclear and cytoplasmic fraction of BV2 microglia cells, respectively. As expected, all the miR-181 family members were found to be preferentially co-precipitated in the cytoplasm, but not in the nucleus (Fig. 3j, k). Taken together, these results demonstrate that lincRNA-p21 physically associates with the miR-181 family and may function as a ceRNA.

### The miR-181 family mediates lincRNA-p21-induced microglial activation

**Fig. 4:**
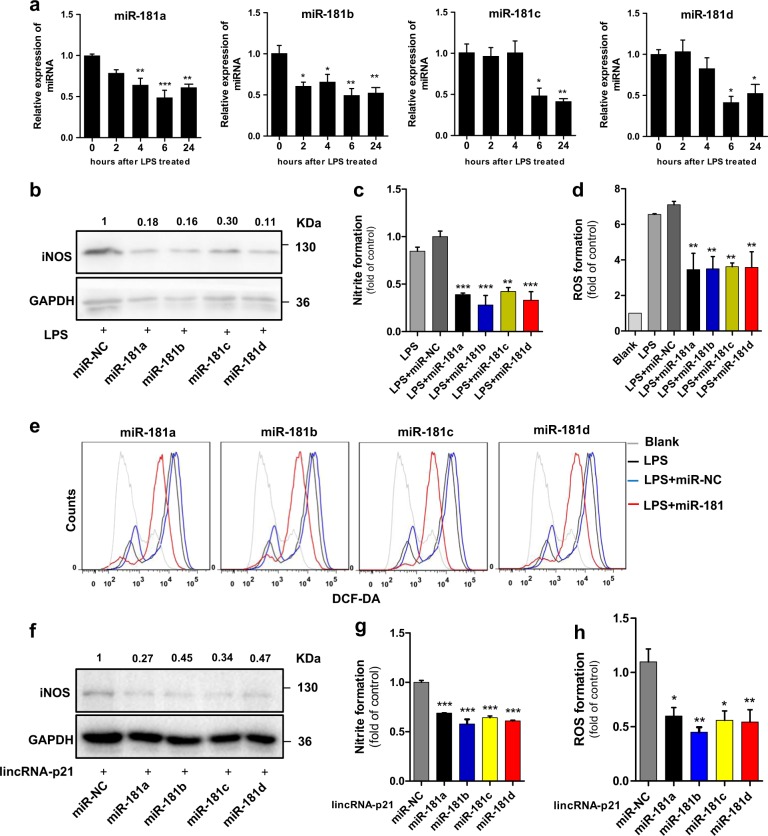
miR-181 family mediates lincRNA-p21-induced microglial activation. **a** BV2 microglia cells were treated with LPS and expression levels of miR-181a, b, c, d were analysed by qRT-PCR. **b**–**d** Effect of miR-181 family overexpression on LPS-induced microglial activation. BV2 microglia cells were transfected with miR-control or miR-181a, b, c, d mimics, and exposed to LPS for 12 h. Cells were harvested and assessed for iNOS expression (**b**), NO production (**c**), and ROS formation (**d**). **e** Representative images of ROS formation by flow cytometry using DCFH-DA dye. **f–h** Effect of miR-181 family overexpression on lincRNA-p21-induced microglial activation. BV2 microglia cells were co-transfected with miR-control or miR-181a, b, c, d mimics in the presence of lincRNA-p21-overexpressing vector. Cells were harvested and assessed for iNOS expression (**f**), NO production (**g**), and ROS formation (**h**). Data are expressed as mean ± SEM (*n* = 3). *P < 0.05, **P < 0.01 and ***P < 0.001 versus control using one-way analysis of variance ANOVA followed by Bonferroni test

We next validated the role of miR-181 family in lincRNA-p21-induced microglial activation. As shown in Fig. 4a, miR-181a/b/c/d gradually decreased during LPS-induced microglial activation, inversely correlating with the expression of lincRNA-p21. We then transfected BV2 microglia cells with miR-181a/b/c/d mimics, respectively, and treated with LPS. All the miR-181 members attenuated microglial activation with downregulation of iNOS, NO and ROS (Fig. 4b–e, Supplementary Fig. 4a). Conversely, transfection of miR-181a/b/c/d inhibitors further enhanced the activating effect of LPS on microglia (Supplementary Fig. 4b–f). Taken together, these experiments demonstrate that all the miR-181 members are involved in the regulation of microglial activation.

Next, we employed rescue experiments by transiently co-transfecting miR-181a/b/c/d mimics and lincRNA-p21-overexpressing vector into BV2 microglia cells, respectively. Phenotype analysis showed that all the miR-181 members prevented the activation of microglia cells resulting from the overexpression of lincRNA-p21, with a reduction of iNOS, NO and ROS (Fig. 4f–h). These data suggested that the functional role of lincRNA-p21 during microglial activation at least partially via the competitive binding of miR-181 family.

### PKC-δ functions as a specific target of miR-181 family in microglial activation

**Fig. 5:**
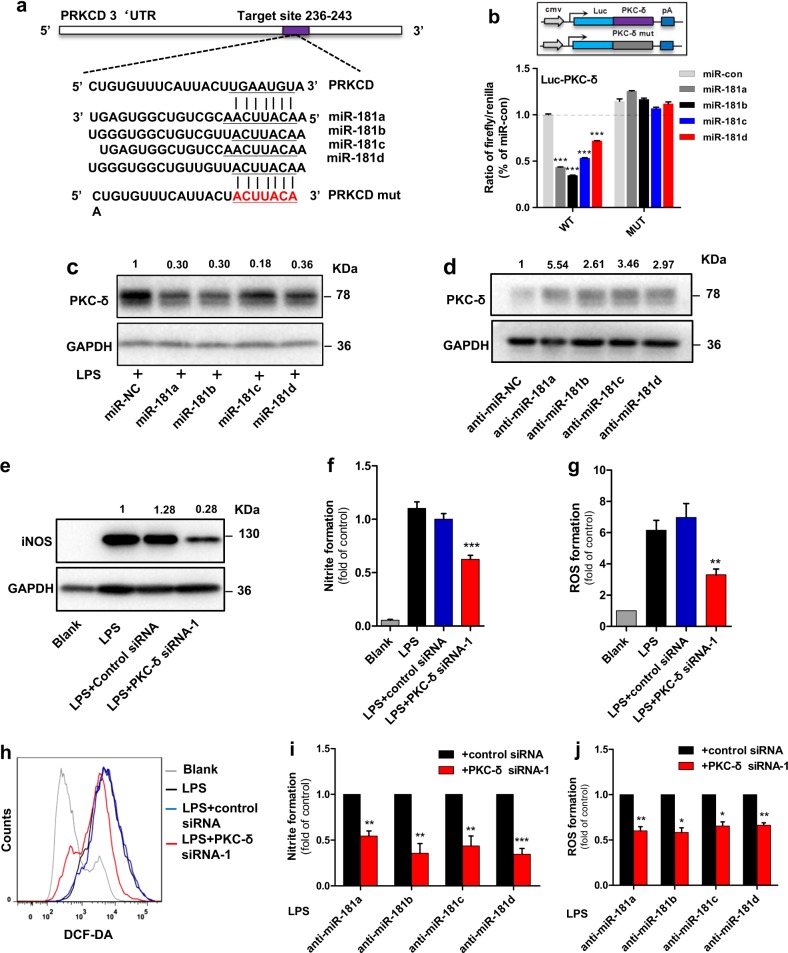
PKC-δ functions as a specific target of miR-181 family in microglial activation. **a** Putative miR-181 family binding site in PKC-δ gene PRKCD. The potential complementary residues are underlined and mutated bases are indicated in red. **b** Upper panel, schematic representation of pmirGLO-based luciferase reporter plasmid containing wild-type PKC-δ (Luc-PKC-δ-wt) and a mutant reporter construct (Luc-PKC-δ-mut). Lower panel, relative luciferase activity of the luciferase constructs of Luc-PKC-δ-wt or Luc-PKC-δ-mut co-transfected with miR-control/miR-181 family in HEK293 cells. A representative experiment with triplicates of two independent experiments is shown. Data are expressed as mean ± SEM of triplicate wells. **P < 0.01, ***P < 0.001 in one-way analysis of variance ANOVA followed by Bonferroni test. **c**, **d** Western blotting of PKC-δ in lysates of BV2 microglia cells tranfected with miR-control/miR-181a, b, c, d mimics (**c**); or anti-miR-control/anti-miR-181 a, b, c, d (inhibitors) (**d**) in the presence of LPS or not. (*n* = 3). **e**–**g** Effect of PKC-δ knockdown on LPS-induced microglial activation. BV2 microglia cells were transfected with control siRNA or PKC-δ specific siRNA-1, followed by the treatment of LPS for 12 h. Cells were harvested and assessed for iNOS expression (**e**), NO production (**f**), and ROS formation (**g**). Data are expressed as mean ± SEM (*n* = 3). **P < 0.01 and ***P < 0.001 versus control using one-way analysis of variance ANOVA followed by Bonferroni test. **h**, **i** Effect of PKC-δ knockdown on microglial activation resulting from miR-181 inhibition. BV2 microglia cells were co-transfected with anti-miR-control or anti-miR-181a, b, c, d (inhibitors) in the presence of PKC-δ specific siRNA-1, followed by the treatment of LPS for 12 h. Cells were harvested and assessed for NO production (**h**) and ROS formation (**i**). Data are expressed as mean ± SEM (*n* = 3). *P < 0.05, **P < 0.01 and ***P < 0.001 versus control using two-tailed unpaired Student’s *t*-test

To identify the target of lincRNA-p21/miR-181 family ceRNA network, we focused on PKC-δ, which has been reported to be the direct target of miR-181 family^24^. Indeed, dual-luciferase assays showed that all the miR-181 family members could greatly reduce the luciferase activities of Luc-PKC-δ-wt but not Luc-PKC-δ-mut (Fig. 5a, b). Ectopically expressed miR-181a/b/c/d led to a reduction of LPS-induced PKC-δ (Fig. 5c), while administration of miR-181a/b/c/d inhibitors could further increase the expression of PKC-δ (Fig. 5d). These results suggest that PKC-δ is a specific target of miR-181 family.

Previous studies have shown that PKC-δ activation contributes to immune signaling events that drive microglial neurotoxicity^25,26^. Indeed, we found increased PKC-δ in LPS-treated BV2 microglia cells (Supplementary Fig. 5d). knockdown of PKC-δ using siRNA resulted in a reduction of iNOS, NO and ROS formation upon LPS treatment (Fig. 5e–h, Supplementary Fig. 5a, b), while overexpressed PKC-δ had an opposing effect (Supplementary Fig. 5c, f, g). We further found that the activating effect of miR-181a/b/c/d inhibition was attenuated by knockdown of PKC-δ simultaneously (Fig. 5i, f). These results demonstrate that the inhibitory effect of miR-181a/b/c/d on microglial activation is due, at least in part, to its negative regulation of PKC-δ expression.

### p53-induced lincRNA-p21 formes a double-negative feedback loop with the miR-181 family/PKC-δ and synergistically promotes activation of microglia

**Fig. 6:**
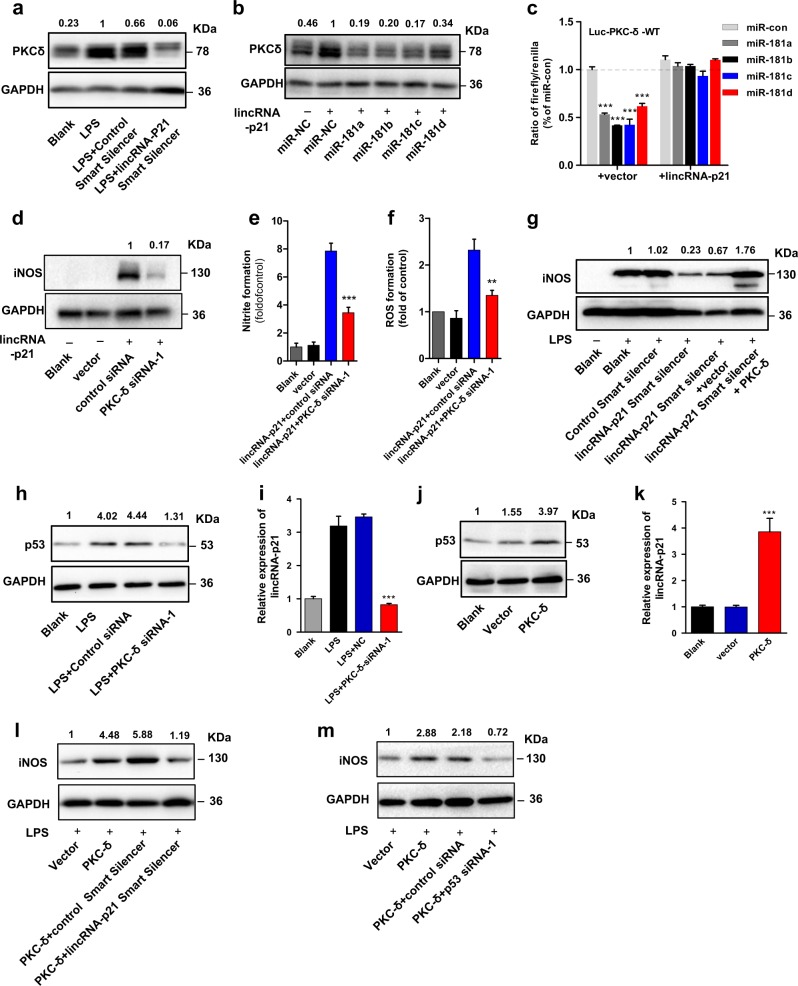
p53-induced lincRNA-p21 formes a double-negative feedback loop with miR-181 family/PKC-δ and synergistically promotes microglial activation. **a** Western blotting analysis of PKC-δ levels in BV2 microglia cells following tranfecction of cells with lincRNA-p21 Smart Silencer or control Smart Silencer in the presence of LPS for 12 h. (*n* = 3). **b** Western blotting analysis of PKC-δ levels in BV2 microglia cells following tranfecction of cells with miR-control/miR-181a, b, c, d mimics in the presence of lincRNA-p21-overexpressing vector. (*n* = 3). **c** Relative luciferase activity of the luciferase constructs of Luc-PKC-δ-wt co-transfected with miR-control/miR-181 family in the presence of either lincRNA-p21-overexpressing vector or control vector in HEK293 cells. A representative experiment with triplicates of two independent experiments is shown. Data are expressed as mean ± SEM of triplicate wells. *P < 0.05, **P < 0.01 in one-way analysis of variance ANOVA followed by Bonferroni test. **d**–**f** Effect of PKC-δ knockdown on lincRNA-p21-induced microglial activation. BV2 microglia cells were co-transfected with control siRNA or PKC-δ siRNA-1 in the presence of lincRNA-p21-overexpressing vector, followed by the treatment of LPS for 12 h. Cells were harvested and assessed for iNOS expression (**d**), NO production (**e**), and ROS formation (**f**). Data are expressed as mean ± SEM (*n* = 3). *P < 0.05, **P < 0.01 versus control using two-tailed unpaired Student’s t-test. **g** BV2 microglia cells were co-transfected with lincRNA-p21 Smart Silencer/control Smart Silencer or PKC-δ-overexpressing vector/control vector, followed by the treatment of LPS for 12 h and assessed for iNOS expression. (*n* = 3). **h**, **i** BV2 microglia cells were transfected with control siRNA or PKC-δ siRNA-1 in the presence of LPS for 12 h, followed by western blotting to assess p53 expression (**h**) and qRT-PCR to detect lincRNA-p21 levels (**i**). **j**, **k** BV2 microglia cells were transfected with PKC-δ-overexpressing vector or control vector, followed by western blotting to assess p53 expression (**j**) and qRT-PCR to detect lincRNA-p21 levels (**k**). Data are expressed as mean ± SEM (*n* = 3). ***P < 0.001 versus control using one-way analysis of variance ANOVA followed by Bonferroni test. **l** BV2 microglia cells were co-transfected with PKC-δ-overexpressing vector/control vector or lincRNA-p21 Smart Silencer/control Smart Silencer, followed by the treatment of LPS for 12 h and assessed for iNOS expression (*n* = 3). **m** BV2 microglia cells were co-transfected with PKC-δ-overexpressing vector/control vector or p53 siRNA/control siRNA, followed by the treatment of LPS for 12 h and assessed for iNOS expression (*n* = 3). All the images are representative immunoblots from three separate experiments

Because lincRNA-p21 shares the regulatory miR-181a/b/c/d with PKC-δ, we wondered whether lincRNA-p21 could modulate PKC-δ expression. Using expression analysis, we found that the depletion of lincRNA-p21 decreased PKC-δ transcript and protein levels in BV2 microglia cells upon LPS treatment (Fig. 6a; Supplementary Fig. 6a–c). Overexpression of lincRNA-p21 increased PKC-δ expression (Fig. 6b; Supplementary Fig. 6d), which was abolished by ectopic expression of miR-181a/b/c/d, respectively (Fig. 6b). To further determine whether lincRNA-p21 could protect PKC-δ from miR-181 family-mediated repression, we co-transfected HEK293 cells with lincRNA-p21-overexpressing vector and Luc-PKC-δ-wt in the presence of miR-181a/b/c/d mimics or NC miRNA, respectively. We found that ectopic expression of lincRNA-p21 efficiently abolished the miR-181a/b/c/d-induced reduction of luciferase activities of Luc-PKC-δ-wt (Fig. 6c). These results indicate that lincRNA-p21 may act as endogenous sponge ‘antagomir’ of miR-181 family, modulating PKC-δ indirectly.

Furthermore, to evaluate whether PKC-δ could mediate the functional role of lincRNA-p21, we knocked down endogenous PKC-δ and overexpressed lincRNA-p21 simultaneously, and found that the lincRNA-p21-induced iNOS, NO and ROS formation were abrogated in BV2 microglia cells in the absence of PKC-δ (Fig. 6d–f). Moreover, ectopic expression of PKC-δ significantly attenuated the reduction of iNOS expression resulting from lincRNA-p21 knockdown in response to LPS treatment (Fig. 6g). Collectively, these results indicate that lincRNA-p21 targets miR-181 family and PKC-δ in the cascade of events leading to microglial activation.

More interestingly, previous reports have shown an association between PKC-δ and p53^27,28^. Indeed, when we depleted PKC-δ with siRNAs, we observed a reduction of p53 and lincRNA-p21 (Fig. 6h, i; Supplementary Fig. 6e), while overexpressed PKC-δ, had an opposing effect (Fig. 6j, k). These data indicate that PKC-δ positively regulates expression of p53 and lincRNA-p21 in BV2 microglia cells. Next, we further ascertained whether p53 or lincRNA-p21 knockdown could rescue the phenotype of PKC-δ-overexpressing cells. As shown in Fig. 6l, m, both the p53 and lincRNA-p21 siRNAs could abolish the increase expression of iNOS in the LPS-treated PKC-δ-overexpressing BV2 microglia cells. In conclusion, considering that lincRNA-p21 also modulate the expression of PKC-δ, we suggest that lincRNA-p21, together with miR-181 family and PKC-δ, form a double-negative feedback loop during the progress of microglial activation.

### The lincRNA-p21-mediated regulatory loop exacerbates LPS-induced inflammation in vivo

**Fig. 7:**
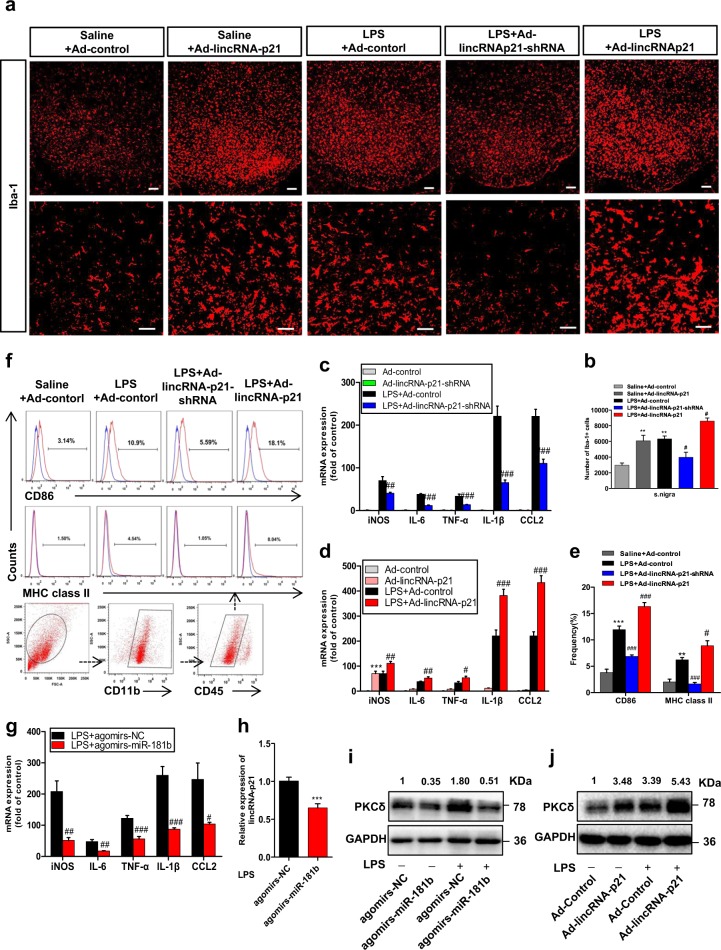
The lincRNA-p21-mediated regulatory loop exacerbated LPS-induced inflammation in vivo. Ad-lincRNA-p21, Ad-lincRNA-p21-shRNA or Ad-control were stereotaxically delivered into the right substantia nigra region of C57BL/6 mice as described in methods. One week later, mice were treated with either saline or LPS (5 mg/kg, i.p.) once for 6 h. **a**, **b** Effect of lincRNA-p21 on microglial activation in the substantia nigra region. **a** Representative immunofluorescence microscopy analysis of Iba-1-positive microglia at the non-injection site in the substantia nigra region. Scale bar = 100 μm (upper panel); 50 μm (lower panel). **b** stereological nigral Iba-1-positive microglia cell counts are shown. Data are expressed as mean ± SEM (*n* = 3 mice per group). **P < 0.01 versus Ad-control group treated with saline; ^#^*P* < 0.05 versus Ad-control group treated with LPS using one-way analysis of variance followed by Bonferroni test. **c**, **d** qRT-PCR analysis of iNOS, IL-6, TNFa, IL-1β and MCP-1 mRNA levels in the ventral mesencephalon infected with Ad-lincRNA-p21-shRNA (**c**), Ad-lincRNA-p21 (**d**) or Ad-control, followed by the treatment of LPS or saline. Data are expressed as mean ± SEM (*n* = 6 mice per group). ***P < 0.001 versus Ad-control group treated with saline; ^#^*P* < 0.05, ^##^*P* < 0.01, ^###^*P* < 0.001 versus Ad-control group treated with LPS using one-way analysis of variance followed by Bonferroni test. **e**, **f** Flow cytometric analysis of expression of CD86 and MHC class II in CD11b^+^/CD45^dim^ population of microglia isolated from the CNS of mice by Percoll gradient method. Quantifications of CD86^+^ and MHC class II^+^ cells in CD11b^+^/CD45^dim^ population of microglia are shown (**e**). Representative flow cytometric analysis of CD11b^+^/CD45^dim^/CD86^+^ cells (upper panel) and CD11b^+^/CD45^dim^/MHC class II^+^ cells (middle panel) are shown (**f**). Data are expressed as mean ± SEM (*n* = 6 mice per group). **P < 0.01, ***P < 0.001 versus Ad-control group treated with saline; ^#^*P* < 0.05, ^###^*P* < 0.001 versus Ad-control group treated with LPS using one-way analysis of variance followed by Bonferroni test. **g**–**i** Mice were injected with agomir-NC or agomir-miR-181b as described in methods, followed by the treatment of either saline or LPS (5 mg/kg, i.p.) for 6 h, and assessed for iNOS, IL-6, TNFa, IL-1β, and MCP-1 mRNA levels (**g**), lincRNA-p21 levels (**h**), and PKC-δ levels (**i**) in the ventral mesencephalon region. Data are expressed as mean ± SEM (*n* = 6 mice per group). ***P < 0.001 versus Ad-control group treated with saline; ^#^*P* < 0.05, ^##^*P* < 0.01, ^###^*P* < 0.001 versus agomir-NC treated with LPS using one-way analysis of variance followed by Bonferroni test. **j** Western blot analysis of PKC-δ levels in the ventral mesencephalon region injected with Ad-lincRNA-p21 or Ad-control, followed by the treatment of LPS or saline. *n* = 4 mice per group

Next, to examine the physiological roles of this lincRNA-p21-mediated regulatory loop in PD pathology, we first utilized the systemic LPS model^25^. At 6 h after injection, we observed a strong induction of lincRNA-p21, PKC-δ and Iba-1, accompanied by a strong reduction of miR-181a/b/c/d expression in the SN tissue (Supplementary Fig. 7a–d). Increased lincRNA-p21 also observed in sorted microglia in Supplementary Fig. 7e. Stereotaxic injections of lincRNA-p21 related adenovirus into the right SN of C57/BL6 mice were then performed. Seven days later, the mice were administered a single dose of LPS (5 mg/kg, i.p.), followed by assessments of the inflammatory response in the SN. As shown in Fig. 7a–c, Supplementary Fig. 7f, delivery of Ad-lincRNA-p21-shRNA in vivo significantly prevented LPS-induced microglial activation as evidenced by a decrease in Iba-1-positive cells and cytokines induction.

Moreover, we further examined the levels of activation markers of microglia isolated using Percoll gradient method followed by flow cytometry analysis. As shown in Supplementary Fig. 7i–k, Ad-lincRNA-p21-shRNA treatment decreased the percentage of CD11b^+^/CD45^hi^ population, while increased the percentage of CD11b^+^/CD45^dim^ population in the presence of LPS. We also found that LPS treatment upregulated the expression of MHC class II and CD86 on resting microglial cells, both of which were significantly prevented by Ad-lincRNA-p21-shRNA treatment (Fig. 7e, f). Reciprocally, stereotaxic injection of adenovirus encoding the full-length lincRNA-p21 (Ad-lincRNA-p21) into the right SN region had an opposing effect (Fig. 7a, b, d–f; Supplementary Fig. 7g, j, k). Confocal immunohistochemical showed showed that Ha-tagged lincRNA-p21 expressed in Iba1-positive microglia cells in the SN region (Supplementary Fig 7l).

We also showed that stereotaxic injection of miR-181b agomir significantly prevented LPS-induced microglial activation as evidenced by decrease in cytokines induction (Fig. 7g, Supplementary Fig. 7h). Additionally, we found that overexpression of miR-181b resulted in a reduction of lincRNA-p21 and PKC-δ (Fig. 7h, i), and overexpression of lincRNA-p21 resulted in a increase of PKC-δ in vivo (Fig. 7j). Taken together, these results suggest that the lincRNA-p21-mediated regulatory loop is critical for mediating LPS-induced microglial activation in vivo.

### LincRNA-p21-mediated inflammation contributes to the death of TH + neurons in the acute MPTP mouse model of PD

**Fig. 8:**
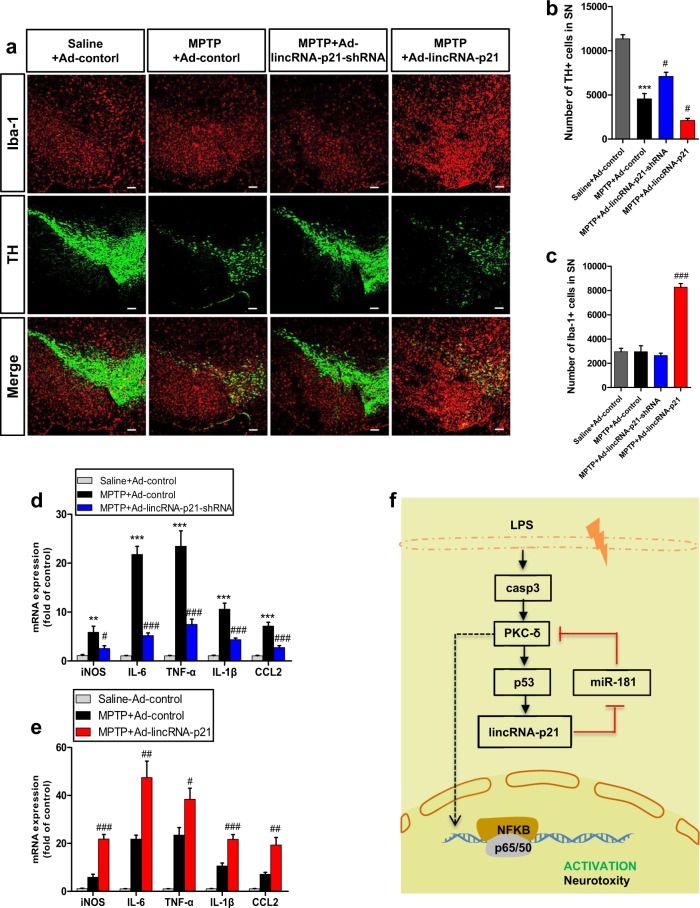
LincRNA-p21-mediated inflammation contributes to the death of TH + neurons in the MPTP model of PD. Ad-lincRNA-p21, Ad-lincRNA-p21-shRNA, or Ad-control were stereotaxically delivered into the right substantia nigra region of C57BL/6 mice as described in methods. One week later, mice received 4 i.p. injections of MPTP-HCl (18 mg/kg free base) at 2 h intervals or equivalent saline injections to generate acute MPTP mouse model of PD. **a** Representative immunofluorescence double staining for Iba1 (microglia, red) and TH (green) at the non-injection site in the substantia nigra region seven days after the last MPTP injection. Scale bar = 100 μm. **b**, **c** Stereological counting of TH-positive cells (**b**) and Iba-1-positive cells (**c**) in the entire substantia nigra are shown. *n* = 3 mice per group. ***P < 0.001 versus Ad-control group treated with saline; ^#^*P* < 0.05, ^###^*P* < 0.001 versus Ad-control group treated with MPTP using one-way analysis of variance followed by Bonferroni test. Two days after the last MPTP injection, total RNA isolated from dissected ventral mesencephalon was subjected to qRT-PCR analysis of iNOS, IL-6, TNFa, IL-1β, and MCP-1 mRNA levels in the ventral mesencephalon infected with Ad-lincRNA-p21-shRNA (**d**), Ad-lincRNA-p21 (**e**) or Ad-control. Data are expressed as mean ± SEM (*n* = 6 mice per group). **P < 0.01, ***P < 0.001 versus Ad-control group treated with saline; ^#^*P* < 0.05, ^##^*P* < 0.01, ^###^*P* < 0.001 versus Ad-control group treated with MPTP using one-way analysis of variance followed by Bonferroni test. **f** A schematic model of lincRNA-p21-related regulatory loop in the modulation of microglial activation

To further substantiate whether lincRNA-p21 signaling during microglial activation could regulate neuroinflammatory responses and promote dopaminergic degeneration in vivo, the well-characterized acute MPTP mouse model of PD was used. AS shown in Supplementary Fig. 8b,c, MPTP treatment induced the expression of lincRNA-p21 and PKC-δ, while suppressed the expression of miR-181a/b/c/d (Supplementary Fig. 8a). Iba-1 immunofluorescence staining seven days after the last MPTP injection revealed that the depletion of lincRNA-p21 could not significantly lower microglial cell density (Fig. 8a, c). However, considering that there was no difference in the numbers of microglia between saline and MPTP-treated group on day seven (Fig. 8a, c), we further analyzed changes in the proinflammatory mediators that are known to be upregulated at around 48 h. As shown in Fig. 8d, delivery of Ad-lincRNA-p21-shRNA in vivo significantly prevented MPTP-induced cytokines induction. Moreover, in Ad-control injected mice, MPTP injections significantly increased the expression of MHC class II and CD86 in the population of CD11b+/CD45dim resting microglial cells. In Ad-lincRNA-p21-shRNA injected mice, however, the MPTP-induced MHC class II and CD86 upregulation was greatly reduced (Supplementary Fig. 8d, e), similar to the results obtained with LPS-treated mice (Fig. 7e, f). Together, these data indicate that lincRNA-p21 is a critical mediator of microglial activation in the acute MPTP mouse model of PD.

Microglial pro-inflammatory response drives the progressive inflammation and exacerbate dopaminergic degeneration over time. Thus, we further examined whether the depletion of lincRNA-p21 exerted a protective effect against MPTP-induced toxicity through inhibition of MPTP-induced microglial activation. TH immunofluorescence staining seven days after the last MPTP injection showed that knockdown of lincRNA-p21 significantly lowered MPTP-induced TH + neurons death, as demonstrated by stereological counts of the TH-positive dopaminergic neurons in the SN (Fig. 8a, b). Conversely, overexpression of lincRNA-p21 by adenovirus further promoted MPTP-induced microglial activation and aggravated TH-positive dopaminergic neuronism lost in the SN (Fig. 8a–c, e, Supplementary Fig. 8d, e). In conclusion, these experiments indicate that lincRNA-p21 promotes microglia-mediated inflammation in the CNS and aggravates TH + neurons degeneration in the MPTP model of PD.

## Discussion

Increasing evidence has linked chronic inflammation mediated by activated microglial cells to a number of neurodegenerative disorders including PD^29^. The activation of microglia is then toxic to neighbouring neurons, leading to further microglial activation and a self-propelling progressive cycle of inflammation and neuron damage^30^. Regardless of the cause of the initial inflammatory stimulus (external stimulus or neuronal death), the microglial pro-inflammatory response to neuron damage can drive the progressive inflammation and cumulative loss of neurons over time. Thus, knowledge of how they are maintained in the sustained activated state and associated continuous neuron damage is of great importance. Recently, studies have yielded important insights into the regulation of inflammation by non-coding RNAs, such as lincRNAs and miRNAs^31,32^. However, such lincRNA and miRNA interactions mediating the key molecular signaling events in microglial activation have not yet been clearly defined. Here, we report that lincRNA-p21 functions as a ceRNA to promote microglial activation and exerts dopaminergic neurotoxicity in both cell culture and murine models of PD.

### LincRNA-p21exerts pro-inflammatory and neurotoxic effects in microglia

lincRNA-p21 is well known to be induced by p53 and exerts diverse functions in multiple diseases^16^. In this study, we focused on the novel role of lincRNA-p21 in promoting inflammation-induced neurotoxicity, which has not yet been clearly illustrated. Indeed, inhibition of endogenous lincRNA-p21 effectively blocked LPS-induced microglial activation (M(LPS) state^33^), while exotic expression of lincRNA-p21 further facilitated microglial activation, skewed the microglia cells toward a pro-inflammatory phenotype, and thus increased the expression of inflammatory mediators and accelerated the death of dopaminergic neurons. Notably, earlier report^34^ demonstrated that methotrexate reduced inflammatory responses in rheumatoid arthritis (RA) patients through the induction of lincRNA-p21. We inferred that this discrepancy could be attributed to the different stimulators and specific disease context used in the two studies^35,36^.

Interestingly, a previous study has demonstrated that the maximum microglia activation response occurred 24–48 h after the last injection of MPTP in the mouse SN^37^, as observed for the expression pattern of lincRNA-p21 in the acute MPTP mouse model of PD, indicating a potent critical role for lincRNA-p21 in mediating the microglial activation response and aggravating TH + neuron loss in PD. In agreement with our in vitro data, we found that ablation of lincRNA-p21 limited inflammatory responses in the SN and protected TH + neurons from MPTP-induced toxicity. Thus, strategies to suppress the expression of neurotoxins by interfering with lincRNA-p21 may have therapeutic utility.

### A p53/lincRNA-p21/miR-181 family/PKC-δ dual-negative feedback loop mediates inflammatory responses of microglia

The present studies demonstrate a potent pro-inflammatory activity of lincRNA-p21 in microglia. We propose that this pro-inflammatory activity is mediated by a p53/lincRNA-p21/miR-181 family/PKC-δ dual-negative feedback loop that further augments this effect (Fig. 8f). It is known that positive feedback loops tend to amplify a response and commitment to a self-sustained mode that is autonomous to the original stimulus^38^. Thus, we propose that this lincRNA-p21-mediated regulatory loop facilitates the conversion of microglia from a steady state to a pro-inflammatory state, eliciting rapid response to the changes in their surroundings in the CNS.

Mechanistically, we provide evidences that lincRNA-p21 serves as an endogenous miRNA sponge for the miR-181 family, indirectly upregulating PKC-δ, and then inducing microglial activation during inflammation. In our in vitro system and in vivo models of neuroinflammation, we observed that overexpression of lincRNA-p21 was sufficient to increase PKC-δ and induce microglial activation. Remarkably, this role depends on the competitive binding of miR-181 family, indicating that lincRNA-p21 functions as a ceRNA. Reduction of PKC-δ or ectopic expression of all the miR-181 family members severely compromises the pro-inflammatory activity of lincRNA-p21. It is widely recognized that lincRNA-p21 tends to act as a downstream effector of p53-dependent transcriptional pathways, regulating cell proliferation^39^, apoptosis^16^, and somatic cell reprogramming^40^. Of interest, we also showed that lincRNA-p21 was functionally dependent on p53 during the regulation of microglial activation, consistent with the important role of p53 in the regulation of chronic inflammation^41^. Moreover, considering the association between PKC-δ and p53, we wondered whether PKC-δ could, in turn, affect the expression of p53/lincRNA-p21 in turn. Notably, we showed that PKC-δ induction could further increase the expression of p53 and lincRNA-p21 in microglia cells, thus ensuing LPS-induced p53/lincRNA-p21 expression. Therefore, we conclude that lincRNA-p21, together with miR-181 family and PKC-δ, forms a regulatory circuit consisting of autoregulatory and dual-negative feedback loops during the progression of microglial activation. Collectively, our data identify a novel model of lincRNA-miRNA interactions during microglia activation. Considering the multiple targets of miRNAs, we also hypothesize that there may be many other lincRNAs that function as ceRNAs to regulate the expression of key genes in microglia cells^42^. Thus, the identification of these ceRNAs may improve our understanding of immune and inflammatory alterations and many related diseases, such as PD.

Based on our data, we propose a model depicting a pivotal role for lincRNA-p21 in the regulation of inflammation-induced neurotoxicity. We identify a regulatory circuit, composed of p53, lincRNA-p21, the miR-181 family and PKC-δ, which mediates a regulatory signal that is critical for eliciting innate immune responses in microglia. Considering that most of our findings were obtained using a single BV2 microglia cell line, further validation in primary microglia cells and other disease models is urgently needed in our future work. These results of such analyses might lead to the development of potential therapeutic agents for the treatment of neuroinflammatory diseases in the central nervous system.

## Method

### Reagents

DMEM, DMEM/F12, fetal bovine serum (FBS), penicillin, streptomycin and other cell culture reagents were purchased from Invitrogen (Carlsbad, CA, USA). LPS (from *Escherichia coli*, serotype 026:B6; Sigma-Aldrich, St. Louis, MO), MPTP (Sigma-Aldrich, St. Louis, MO), lipoteichoic acid (LTA, from Staphylococcus aureus, Sigma-Aldrich, St. Louis, MO), PamC3sk4 (R&D Systems) and mouse interferon-gamma (IFN-γ, Thermo Fisher Scientific) were used in this study. pcDNA3.1-licRNA-p21 and PKC delta WT (Addgene plasmid # 16386)^43^ were kind gifts from Nadya Dimitrova and Bernard Weinstein, respectively.

### Cell cultures and treatment

We used murine microglial BV2 cells to examine the molecular mechanism of microglia activation. BV2 cells were grown and routinely maintained in high-glucose DMEM (Invitrogen, Carlsbad, CA, USA) supplemented with 10% heat-inactivated FBS (Gibco, Grand Island, NY, USA), penicillin (100 IU/ml) and streptomycin (100 mg/ml) at 37 °C in a humidified incubator with 5% CO2. FBS was reduced to 5% when the experiments were performed. LPS (1 μg/ml), LTA (100 µg/ml), PamC3sk4 (100 ng/ml), and IFN-γ (10 ng/ml) were used in this study.

### Conditioned media (CM) assays

SH-SY5Y cells were obtained from the Central Laboratory of Nanfang Hospital (Guangzhou, China). Cells were maintained in DMEM/F12 (Invitrogen, Carlsbad, CA, USA) supplemented with 10% FBS (Gibco, Grand Island, NY, USA) as described^44^. A CM transfer system was used to determine the impact of LPS-treated BV2 cells on SH-SY5Y cells^45^. CMs from control and lincRNA-p21-overexpressed BV2 cells were filtered through 0.45-μm filters and frozen at −80 °C. SH-SY5Y cells were cultured with CMs (diluted with fresh medium at a 1:1 ratio) for 24 h and apoptosis assays were analyzed by flow cytometry (Becton-Dickinson Immunocytometry Systems, San Jose, CA) using an Annexin V-FITC and propidium iodide (PI) kit (Invitrogen, Carlsbad, CA, USA).

### Flow cytometry assay

Surface antigens changes in primary mouse microglia were assayed by flow cytometry, as described^46^. Briefly, the purified cells were incubated with 5 μl of anti-mouse Fc receptor block antibody (CD16/CD32, eBioscience) for 15 min on ice. The cells were then surface-stained with fluorescein isothiocyanateconjugated anti-CD11b-APC-eflour780, anti-CD45-PE-Cyanine7, anti-MHC class II(I-A/I-E)-PE, anti-CD86(B7-2)-PE-Cyanine5, and anti-CD206(MMR)-APC (eBioscience, San Diego, CA) for 20 min on ice in the dark. Data were acquired with an LSRII cytometer (BD Biosciences, San Jose, CA) and analyzed with the FlowJo software program (TreeStar, Ashland, OR). Positive and negative gates were drawn based on the intensity of an unstained control sample.

### Nitrite assay

The expression of nitrite (NaNO_2_), a stable end product resulting from the reaction of NO with molecular oxygen, was measured in supernatants collected from treated and untreated microglia using a Griess reagent system (Promega, Madison, WI, USA) according to the manufacturer’s instructions. A synergy Mx fluorescence plate reader (Bio-Tek Instruments) was used to detect the absorbance at 550 nm. The concentration of nitrite was calculated from a standard curve using sodium nitrite solution.

### Assessment of ROS production

The levels of intracellular ROS was determined using DCFH-DA dye (Sigma-Aldrich, St. Louis, MO) as described previously^47^. Briefly, BV2 cells were plated in 6-well polystyrene culture plates at a density of 2 × 105 cells. After LPS treatment, the cells were incubated with 10 µM DCFH-DA dye for 30 min in the dark, in a conventional incubator (37 °C, 5% CO2) and washed three times with PBS. ROS were detected immediately by flow cytometry (BD Biosciences, San Jose, CA) using the FL1 green fluorescence channel. Data was collected as the mean fluorescence intensity and analyzed using the FlowJo software program (TreeStar, Ashland, OR).

### RNA extraction and quantitative RT–PCR analysis

Total RNA was extracted from cells and mouse brain tissue samples using RNAiso Plus (Takara, Japan). A NanoDrop ND-2000 Spectrophotometer (Thermo Fisher Scientific, Inc., Wilmington, DE, USA) was used to quantify the RNA concentration. Stem-loop qRT-PCR for mature miRNA expression was performed. For assessments of mRNA expression, 1 μg total RNA was used to synthesize the complementary DNA using a PrimeScript™ RT reagent kit with gDNA Eraser (Takara, Japan). cDNA products were then diluted 1:10 in ddH2O. qRT-PCR was performed using SYBR® Premix Ex Taq™ II and a CFX96 real-time PCR system (Bio-Rad, Hercules, USA). U6 snRNA (for miRNAs) or β-actin expression (for mRNAs) were used as control for normalization and the relative gene expression levels were calculated using the 2^−(ΔΔCt)^ method^48^. U6 snRNA premier set was purchased from RiboBio (Guangzhou, China). Other primer information provided in Supplementary Table 1.

### Western blotting

Brain tissues and microglial cell lysates were prepared in standard RIPA buffer (Beyotime, Jiangsu, China) containing 1 × protease and 1 × phosphatase inhibitor cocktails (Selleck Chemicals, Shanghai, China). Western blotting was carried out as previously described^49^. Briefly, equal amounts of the proteins were loaded on 10% or 15% SDS-PAGE gels for separation under reducing conditions, followed by blotting of the protein on a PVDF membrane (Immobilon-P, Millipore) in a Mini Trans-Blot electrophoretic transfer cell (Bio-Rad). Membranes were then blocked with 5% non-fat dry milk in TBS with 0.1% Tween and probed with appropriate primary antibodies against PKC-δ (1:1,000, Santa Cruz Biotech, sc-9379), iNOS (1:1000, Santa Cruz Biotech, sc-650), p53 (1:1000, Cell Signaling, #2524), Ago2 (1:1000, Abcam, ab32381), histone H3 (1:1000, Abcam, ab4729), and GAPDH (1:5000, Bioworld, AP0063) overnight at 4 °C. HRP-conjugated secondary antibodies (goat anti mouse/rabbit IgG 1:5000) were used for antibody detection with the immobilon ECL kit (Merck Millipore) in a ChemiDoc XRS + system (Bio-Rad) and band intensities were quantified using ImageJ software (US National Institutes of Health)^50^. Full blots are presented in Supplementary Fig 9.

### Nuclear and cytoplasmic fractionation

To determine the subcellular distributions of lincRNA-p21, miR-181 family and Ago2, we performed subcellular fractionation using a PARIS kit (Ambion, Austin, Texas), according to the manufacturer’s protocols. After adding an exogenous spike-in of cel-mir-39 (GenePharma, Shanghai, China) to each sample as a normalizer for the qPCR analysis, RNA was extracted using RNAiso Plus (Takara, Japan), and equal volumes of ddH2O were used to resuspend the resulting RNA pellets to obtain cell-equivalent concentrations^51^. The efficiency of the nuclear and cytoplasmic fractions was determined by comparing the abundance of nuclear RNA (U2 snRNA) or cytoplasmic mRNA (18S rRNA) using qRT-PCR. It was also assessed by quantifying the known cytoplasmic protein GAPDH and nuclear protein histone H3.

### Dual-luciferase assay

The sequence from wild-type or mutants 3′-UTR of PRKCD and lincRNA-p21 containing the common miR-181 family binding site were synthesized and cloned downstream of the luciferase reporter gene into the pmirGLO vector (Promega, Madison, WI, USA) using the SacI and SalI restriction enzymes. For mutated 3′-UTRs, six or eight bases of each seed region were replaced with complementary bases. For the luciferase reporter assays, HEK293T cells were co-transfected with the indicated luciferase constructs with synthetic miRNA mimics (Ribobio, Guangzhou, China), and luciferase activity was measured using a dual reporter luciferase assay kit (Promega, Madison, WI, USA), according to the manufacturer’s protocol. Renilla luciferase activity was normalized to the corresponding firefly luciferase activity and plotted as a percentage of the controls.

### RNA-binding protein immunoprecipitation (RIP) assay

For the anti-Ago2 RIP experiments, a EZ-Magna RIP™ RNA-Binding Protein Immunoprecipitation Kit (Millipore, USA) and mouse anti-Ago2 antibody (Abcam, ab32381) were used according to the manufacturer’s instructions^52^. Briefly, BV2 cells were harvested and lysed with the buffer provided in the kits and sonicated six times for 30 s to facilitate lysis. Immunoprecipitation was performed using anti-Ago2 or control IgG (negative control) and snRNP70 (positive control). The RNA fraction isolated was then treated with RNAiso Plus (Takara, Japan) for further purification and qRT-PCR analysis. Total RNAs from cell lysate (Input) were detected simultaneously as controls.

### Biotin RNA pull-down assay

The RNA pull-down assay was performed as previously described^53^ with modifications. In brief, the DNA fragment covering the entire mouse lincRNA-p21 sequence was PCR-amplified from vector pcDNA3.1-lincRNA-p21 using T7-containing primers (lincRNA-p21 primers forward 5′-ATATCTCCAAAGACCCAGGGC-3′, reverse 5′-ACACGTGTGTATGATTGTCTGTGC-3′) and then cloned into pGEM-T (Promega, Madison, WI, USA). After linearization with the restriction enzyme NotI, the full-length of mouse lincRNA-p21 was labeled with biotin using Biotin RNA Labeling Mix (Roche) and T7 RNA polymerase (Roche), then treated with RNase-free DNase I (Roche), and purified with a RNeasy Mini Kit (Qiagen, Valencia, CA). The probes were then incubated with streptavidin-coated magnetic beads (Invitrogen, Carlsbad, CA, USA) at 4 °C over-night to generate probe-coated magnetic beads. Next, 1 mg whole-cell lysates from BV2 cells were incubated with 3 μg of probe-coated beads for 1 h at room temperature, and the isolated complexes were used either for extraction of the RNA for qRT-PCR or for western blotting according to standard procedures.

### Oligos and plasmid transfection

siRNA oligos targeting mouse lincRNA-p21 (#1 UGAAAAGAGCCGUGAGCUATT, #2 AAAUAAAGAUGGUGGAAUGTT), mouse p53 (#1 AGAAGAAAAUUUCCGCAAATT), mouse PKC-δ (#1 GGAUGUGCAAAGAGAAUAUTT, #2 GGAGUGACAUCCUAGACAATT) and negative control (NC) siRNA (UUCUCCGAACGUGUCACGU) were synthesized by GenePharma biotechnology (Shanghai, China). LncRNA Smart Silencer, synthesized by RiboBio (Guangzhou, China), was also used to knock down the expression of lincRNA-p21. MiR-181a, b, c, d mimics, inhibitors and negative controls (mimic control, inhibitor control) were purchased from RiboBio (Guangzhou, China). These oligonucleotides were all transfected into BV2 cells using Lipofectamine 2000 (Invitrogen) at a final concentration of 100 nM. For overexpression, pcDNA3.1-licRNA-p21 and PKC delta WT vector were transfected into BV2 cells using Lipofectamine 2000, according to the manufacturer’s instructions.

### Adenoviral constructions and purification

The lincRNA-p21 related adenoviruses were constructed using the Adeno-XTM expression system (Clontech). Briefly, full length of mouse lincRNA-p21 was subcloned into adenoviral shuttle vector pDC315 (Microbix Biosystems, Ontario, Canada) to generate pDC315-lincRNA-p21 (Ad-lincRNA-p21). LincRNA-p21 shRNA harboring lincRNA-p21 siRNA #1 (Ad-lincRNA-p21-shRNA) was designed, synthesized and inserted into pDC315-U6-shRNA. Adenoviruses were then produced by co-transfecting HEK293T cells with each adenoviral construct together with the packaging vectors pBHGloxdelE13cre (Microbix Biosystems, Ontario, Canada) using Lipofectamine 2000 (Life Technologies), according to the instructions of the manufacturer. Ad-lincRNA-p21, Ad-lincRNA-p21-shRNA and Ad-control (used as control) were then amplified and purified using the ViraTrap^TM^ adenovirus purification kit (Biomiga Inc., San Diego, Calif., USA). All the adenoviruses were tagged with HA epitope.

### Animals and treatment

C57BL/6 mice (male, 6–8 weeks) were purchased from the Laboratory Animal Centre of Southern Medical University. Animal care and procedures were carried out with the approval of the Southern Medical University Ethics Committee and strictly complied with the National Institutes of Health (NIH) Guide for the Care and Use of Laboratory Animals. Two animal models were used. The well-characterized acute MPTP mouse model of PD^25^ was used for the neuroinflammation and neuroprotection studies because the kinetics of microglial neuroinflammation is well characterized in this model. The mice from the MPTP treatment group received 4 i.p. injections of MPTP-HCl (18 mg/kg free base) at 2-h intervals and control mice received equivalent saline injections. The mice were killed at indicated time points: 0 (immediately after the last MPTP injection), 1, 2, 4, and 7 days after the last MPTP injection. The nigral neuroinflammatory response was also studied using the systemic LPS injection model, which was used to induce chronic neuroinflammation and delayed dopaminergic degeneration in mice^54^. The mice received a single injection of LPS (5 mg/kg, i.p.) or equivalent injections of saline. The mice were killed 6 h later.

### Stereotaxic surgery

Stereotaxic surgery was carried out with a stereotaxic frame (Stoelting, Wood Dale, IL, USA) and a 5 µl Hamilton syringe fitted with a pulled glass capillary tube. After anesthetization, the mice were head immobilized in a flat-skull position for stereotaxic surgery. Adenoviruses (2 μl; 1 × 10^10^ IFU/μl per construct) and micrON^®^ miRNA agomir (NC or miR-181b, 1 nmol/2 μl per mouse) from RiboBio were stereotaxically delivered into the right substantia nigra region (AP: 3.1 mm, ML: 1.2 mm, DV: 5.1 mm from bregma) as previously reported^26^. One week later, the mice were treated with either saline or MPTP/LPS as described above. All mice were killed at the indicated time points.

### Immunofluorescence and image analysis

Immunofluorescence was performed as described previously^45,55^. Briefly, the animals were anesthetized and transcardially perfused with PBS followed by ice-cold 4% paraformaldehyde. Brains were postfixed in the same fixative for 2 days at 4 °C and then cryoprotected in 30% sucrose for another 2 days at 4 °C. Series of mesencephalic coronal sections (12 μm) were collected using a freezing microtome (Leica) and mounted on PDL-coated slides. Sections were incubated with the indicated primary antibodies. For Iba-1 immunostaining, sections were probed with rabbit anti-Iba1 (1:500, Wako Chemicals, Japan) over-night at 4 °C and then incubated with Cy3-conjugated goat anti-rabbit IgG (1:300, Servicebio, Wuhan, China). To analyze the interplay between Iba-1 and TH cells, double-labeling immunofluorescence was performed. Brain sections were incubated with mouse anti-TH (1:250, Millipore, MA, USA) and rabbit anti-Iba1 (1:500, Wako Chemicals, Japan) followed by an incubation with goat anti-mouse IgG conjugated to Alexa Fluor 488 (1:400, Servicebio, Wuhan, China) and Cy3-conjugated goat anti-rabbit IgG (1:300, Servicebio, Wuhan, China), respectively. Mouse anti-HA (1:200, abcam, USA) was used to determine the efficacy of lincRNA-p21 related adenoviruses transduction in vivo. The 4,6–diamidino-2-phenylindole (DAPI, 1:1000, Roche) was used to identify nuclei. Fluorescence images were obtained with a Zeiss LSM 880 (Carl Zeiss, Jena,Germany) laser-scanning microscope and confocal images were acquired and analyzed using a ZEN lite 2012 software. For each animal, eight sections covering the entire antero-posterior ventral mesencephalon were analysed. Total numbers of TH-stained neurons and Iba-1 positive microglia in the substantia nigra region were counted using the Optical Fractionator method with Microbrightfield Stereo-Investigator software (Microbrightfield, VT, USA) as described previously^54,56^.

### Adult microglia isolation and flow cytometry analysis

At 6 h after LPS treatment, microglia cells from the adenovirus injected groups were isolated from whole brain homogenates by Percoll gradient centrifugation according to previous reports^46,57,58^. Briefly, brain tissue was gently dissociated by grinding in a 15-ml dounce homogenizer (Glass Potter, Braun, Melsungen, Germany) containing DNase I (0.025 U ml^–1^ final concentration, Sigma) and passed through 70-μm filtration (BD Biosciences, Boston, MA). After centrifugation, the supernatants were discarded, and the cell pellets were resuspended in 40% Percoll (GE-healthcare, Uppsala, Sweden), followed by spining at 800 g for 30 min with discontinuous Percoll gradients (HBSS/30%/40%/70%). Microglia cells were collected at the 40%/70% interface. Cells were washed and then resuspended in FACS buffer (1X PBS with 2% FBS). Quantification of CD11b + cells with CD45, MHC class II, and CD86 was performed with a FACSCalibur flow cytometer (Becton Dickinson) using standard procedures.

### Statistical analysis

All data are presented as means ± standard error (SEM). Statistical analyses were performed using SPSS version 19 (SPSS, Inc., Chicago, IL, USA). Significant differences between the two groups were carried out using the two-tailed unpaired Student’s *t*-test. Comparisons between multiple groups were performed by one-way or two-way analysis of variance ANOVA with Bonferroni post hoc tests. *P* < 0.05 was considered statistically significant.

## Electronic supplementary material

Supplementary figure legends

supplementary figure 1

supplementary figure 2

supplementary figure 3

supplementary figure 4

supplementary figure 5

supplementary figure 6

supplementary figure 7

supplementary figure 8

supplementary figure 9

supplementary table 1
