# Supplementary material for: A lincRNA-p21/miR-181 family feedback loop regulates microglial activation during systemic LPS- and MPTP- induced neuroinflammation
Source: Cell Death Dis. 2018 Jul 23;9(8):803. doi: 10.1038/s41419-018-0821-5 (PMC6056543; doi:10.1038/s41419-018-0821-5)
Supplement: Supplementary file 1 — Supplementary figure legends [file 41419_2018_821_MOESM1_ESM.docx]

Supplementary figure legends

**Supplementary Figure 1. LincRNA-p21 promotes microglial activation.**

(**a**) BV2 microglia cells were transfected with lincRNA-p21 Smart Silencer or control Smart Silencer and exposed to LPS. LincRNA-p21 levels were analysed by qRT-PCR. Data are expressed as mean ± SEM (n=3). ***P<0.001 versus control using one-way analysis of variance ANOVA followed by Bonferroni test. (**b**) BV2 microglia cells were transfected with lincRNA-p21 siRNA-1, siRNA-2 or control siRNA and lincRNA-p21 levels were analysed by qRT-PCR. Data are expressed as mean ± SEM (n=3). **P<0.01 versus control using one-way analysis of variance ANOVA followed by Bonferroni test. (**c**) BV2 microglia cells were transfected with lincRNA-p21-overexpressing vector or control vector and lincRNA-p21 levels were analysed by qRT-PCR. Data are expressed as mean ± SEM (n=3). **P<0.01 in two-tailed unpaired Student’s t-test. (**d**) Effect of lincRNA-p21 Smart Silencer on LPS-induced IL-6, TNFa, IL-1β and MCP-1 mRNA levels. Effect of lincRNA-p21 siRNA-1 on LPS-induced iNOS expression (**e**), NO production (**g**) and ROS formation (**i**). Effect of lincRNA-p21 siRNA-2 on LPS-induced iNOS expression (**f**), NO production (**h**) and ROS formation (**j**). (**k**) BV2 microglia cells were treated with LTA, PamC3sk4 and IFN-γ and expression levels of lincRNA-p21 were analysed by qRT-PCR. Effect of lincRNA-p21 Smart Silencer on LTA, PamC3sk4 and IFN-γ-induced ROS formation (**l**) and iNOS expression (**m-o**). Data are expressed as mean ± SEM (n=3). *P<0.05, **P<0.01, ***P<0.001 versus control using two-tailed unpaired Student’s t-test or one-way analysis of variance ANOVA followed by Bonferroni test. ##P<0.01, ###P<0.001 versus control group treated with LTA, PamC3sk4 or IFN-γ using one-way analysis of variance followed by Bonferroni test.

**Supplementary Figure2. Knockdown of p53 prevents LPS-induced microglial activation.**

(**a**) BV2 microglia cells were transfected with p53 specific siRNA or control siRNA. 48h after transtection, p53 levels were analysed by Western blotting. (**b**) BV2 microglia cells were co-transfected with p53 specific siRNA or control siRNA and lincRNA-p21 Smart Silencer and treated with LPS. Cells were then harvested and assessed for iNOS expression by western blotting. All the images are representative immunoblots from three separate experiments.

**Supplementary Figure 3. Subcellular distributions of lincRNA-p21, miR-181 family and Ago2.**

(**a**) Representative immunoblots of cytoplasmic protein GAPDH and nuclear protein histone H3. C, cytoplasmic; N, nuclear. (**b**) Representative immunoblots of Ago2 distribution in BV2 microglia cells. T, total; C, cytoplasmic; N, nuclear. (**c**,**d**) qRT-PCR analysis of miR-181 family (**c**) and lincRNA-p21 (**d**) distribution in nuclear and cytoplasmic fractions in BV2 microglia cells treated with LPS or PBS. Data are expressed as mean ± SEM (n=3). **P<0.01 and ***P<0.001 versus control using two-way analysis of variance ANOVA followed by Bonferroni test.

**Supplementary Figure 4. Knockdown of miR-181 family facilitates LPS-induced microglial activation.**

(**a**,**b**) qRT-PCR analysis of miR-181 a, b, c, d levels in BV2 microglia cells following tranfecction of cells with miR-control/miR-181a, b, c, d mimics (**a**); or anti-miR-control/anti-miR-181 a, b, c, d (inhibitors) (**b**). Data are expressed as mean ± SEM (n=3). *P<0.05, **P<0.01 and ***P<0.001 versus control using two-tailed unpaired Student’s t-test. (**c**-**e**) Effect of miR-181 knockdown on LPS-induced microglial activation. BV2 microglia cells were transfected with anti-miR-control or anti-miR-181a, b, c, d (inhibitors), and exposed to LPS for 12h. Cells were harvested and assessed for iNOS expression (**c**), NO production (**d**) and ROS formation (**e**). (**f**) Representative images of ROS formation by flow cytometry using DCFH-DA dye. Data are expressed as mean ± SEM (n=3). *P<0.05, **P<0.01 and ***P<0.001 versus control using one-way analysis of variance ANOVA followed by Bonferroni test.

**Supplementary Figure 5. PKC-δ regulates microglial activation.**

(**a**-**c**) Western blotting analysis of PKC-δ levels in BV2 microglia cells following tranfecction of cells with control siRNA or PKC-δ siRNA-1 (**a**), PKC-δ siRNA-2 (**b**); or control vector/PKC-δ-overexpressing vector (**c**). (n=3). (**d**) Western blotting analysis of PKC-δ expression in BV2 microglia cells treated with LPS for different time points. (n=3). BV2 microglia cells were transfected with control siRNA or PKC-δ siRNA-2, followed by the treatment of LPS for 12h. Cells were harvested and assessed for iNOS expression (**e**). (**f**,**g**) Effect of PKC-δ overexpression on microglial activation. BV2 microglia cells were transfected with control vector or PKC-δ-overexpressing vector for 48h and assessed for NO production (**f**) and ROS formation (**g**, left panel). Representative images of ROS formation by flow cytometry using DCFH-DA dye (**g**, right panel). Data are expressed as mean ± SEM (n=3). *P<0.05, ***P<0.001 versus control vector using one-way analysis of variance ANOVA followed by Bonferroni test.

**Supplementary Figure 6. LincRNA-p21 regulates the expression of PKC-δ.**

(**a**) qRT-PCR analysis of lincRNA-p21 levels in BV2 microglia cells following tranfecction of cells with lincRNA-p21 Smart Silencer or control Smart Silencer. Data are expressed as mean ± SEM (n=3). ***P<0.001 versus control using one-way analysis of variance ANOVA followed by Bonferroni test. (**b**,**c**) Western blotting analysis of PKC-δ levels in BV2 microglia cells following tranfecction of cells with lincRNA-p21 siRNA-1 (**b**), siRNA-2 (**c**) or control siRNA in the presence of LPS for 12h. (n=3). (**d**) Western blotting analysis of PKC-δ levels in BV2 microglia cells following tranfecction of cells with lincRNA-p21-overexpressing vector or control vector. (n=3). (**e**) Western blotting analysis of p53 levels in BV2 microglia cells following tranfecction of cells with PKC-δ siRNA-2 or control siRNA in the presence of either LPS or PBS. (n=3).

**Supplementary Figure 7. LincRNA-p21 regulates microglial activation in vivo.**

(**a**-**c**) C57BL/6 mice were treated with either saline or LPS (5mg/kg, i.p.) once for 6h. Total RNA and protein isolated from dissected ventral mesencephalon were subjected to qRT-PCR analysis of lincRNA-p21 levels (**a**), miR-181a, b, c, d levels (**b**), Iba-1 mRNA levels (**d**) (n=6 mice per group) and western blotting analysis of PKC-δ levels (**c**) (n=4 mice per group). Effect of LPS on the expression of lincRNA-p21 in microglia isolated by Percoll gradient method. Total RNA isolated from microglia was subjected to qRT-PCR analysis of lincRNA-p21 levels (**e**). Three independent experiments were performed, with each group containing pooled cells from four or five mice. Levels of lincRNA-p21 in the ventral mesencephalon of Ad-control, Ad-lincRNA-p21-shRNA (**f**) and Ad-lincRNA-p21 (**g**) groups. (**h**) Levels of miR-181b in the ventral mesencephalon of agomir-NC and agomir-miR-181b groups. Data are expressed as mean ± SEM (n=3 mice per group). *P<0.05, **P<0.01, ***P<0.001 versus control group using two-tailed unpaired Student’s t-test. (**i**) Representative image of mononuclear cells isolated from CNS gated by the expression of CD45 and CD11b. (**j**) Flow cytometric analysis of expression of CD45 in CD11b^+^ population of mononuclear cells isolated from the CNS of mice by Percoll gradient method. Percentages of CD11b^+^/CD45^hi^ cells are shown. Hi, high. (**k**) Quantitative analysis of expression of CD11b^+^/CD45^hi^ cells are shown. Data are expressed as mean ± SEM (n=6 mice per group). *P<0.05 versus Ad-control group treated with saline; #P<0.05, ##P<0.01 versus Ad-control group treated with LPS using one-way analysis of variance followed by Bonferroni test. (**l**) Triple labeling immunofluorescence for HA (Green), Iba1 (Red) and DAPI (Blue) in SN region of mouse brain stereotaxically injected with Ad-lincRNA-p21. Solid arrowheads indicate HA-expressing Iba1-positive microglia. Scale bar, 50 μm.

**Supplementary Figure 8. Relative expression of** **lincRNA-p21, PKC-δ and miR-181 family in the SN of acute MPTP mouse model of PD.**

C57BL/6 mice received 4 i.p. injections of MPTP-HCl (18 mg/kg free base) at 2 h intervals or equivalent saline injections to generate acute MPTP mouse model of PD. Mice were sacrificed at desired timepoints. Total RNA and protein isolated from dissected ventral mesencephalon were then subjected to qRT-PCR analysis of miR-181a, b, c, d levels (**a**), lincRNA-p21 levels (**b**) (n=6 mice per group), and western blotting analysis of PKC-δ levels (**c**) (n=4 mice per group). Data are expressed as mean ± SEM. *P<0.05, **P<0.01, ***P<0.001 versus saline group using one-way analysis of variance followed by Bonferroni test. (**d,e**) Flow cytometric analysis of expression of CD86 and MHC class II in CD11b^+^/CD45^dim^ population of microglia isolated from the CNS of mice by Percoll gradient method two days after the last MPTP injection. Representative flow cytometric analysis of CD11b^+^/CD45^dim^/CD86^+^ cells (upper panel) and CD11b^+^/CD45^dim^/MHC class II^+^ cells (middle panel) are shown (**d**). Quantifications of CD86^+^ and MHC class II^+^ cells in CD11b^+^/CD45^dim^ population of microglia are shown (**e**). Data are expressed as mean ± SEM (n=6 mice per group). ***P<0.001 versus Ad-control group treated with saline; #P<0.05, ##P<0.01, ###P<0.001 versus Ad-control group treated with MPTP using one-way analysis of variance followed by Bonferroni test.

**Supplementary Figure 9.** Uncropped images of blots presented in the main paper are shown.

Molecular weight markers are indicated in kDa.

**Supplementary Table 1.** Primer sequences. All sequences are given 5’ to 3’.
