# Supplementary figures and images for: A lincRNA-p21/miR-181 family feedback loop regulates microglial activation during systemic LPS- and MPTP- induced neuroinflammation
Source: Cell Death Dis. 2018 Jul 23;9(8):803. doi: 10.1038/s41419-018-0821-5 (PMC6056543; doi:10.1038/s41419-018-0821-5)

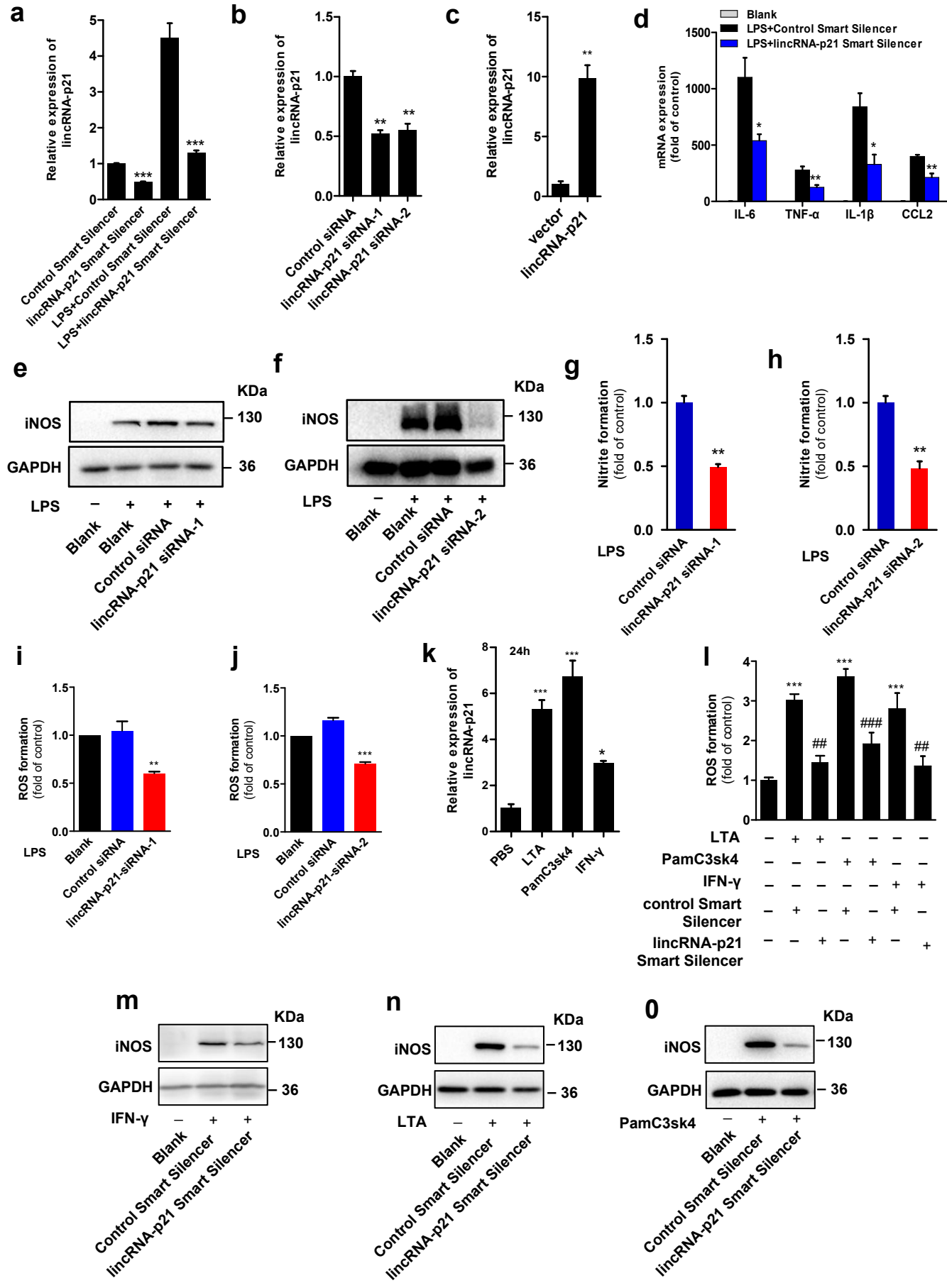

Supplement: Supplementary file 2 — supplementary figure 1 [file 41419_2018_821_MOESM2_ESM.pdf]

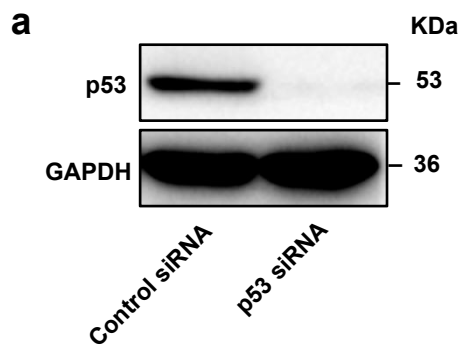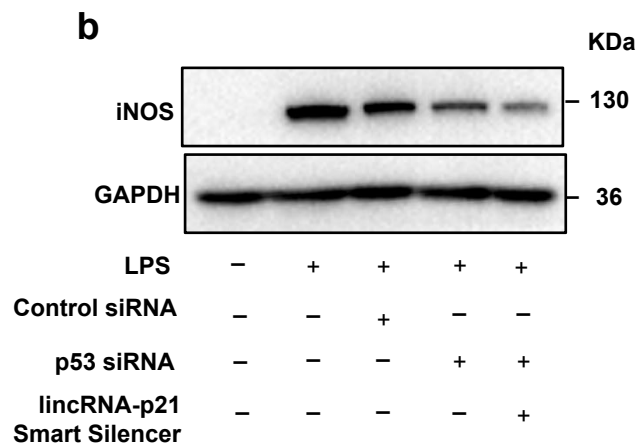

Supplement: Supplementary file 3 — supplementary figure 2 [file 41419_2018_821_MOESM3_ESM.pdf]

**a**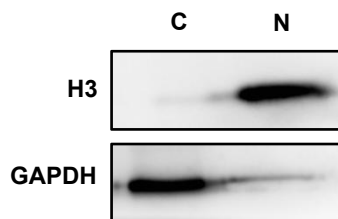**b**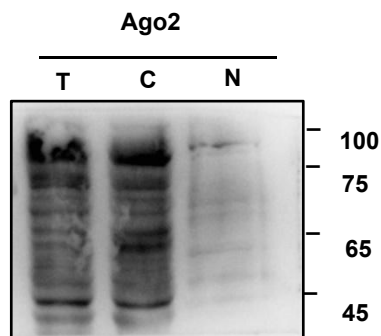**c**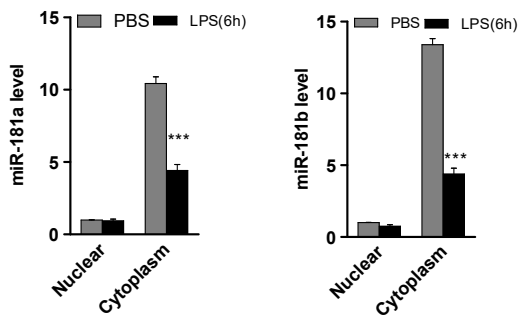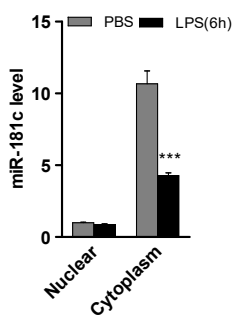**d**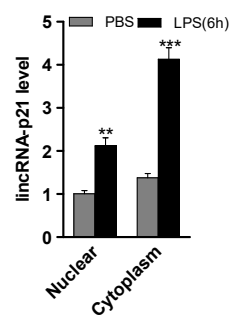

Supplement: Supplementary file 4 — supplementary figure 3 [file 41419_2018_821_MOESM4_ESM.pdf]

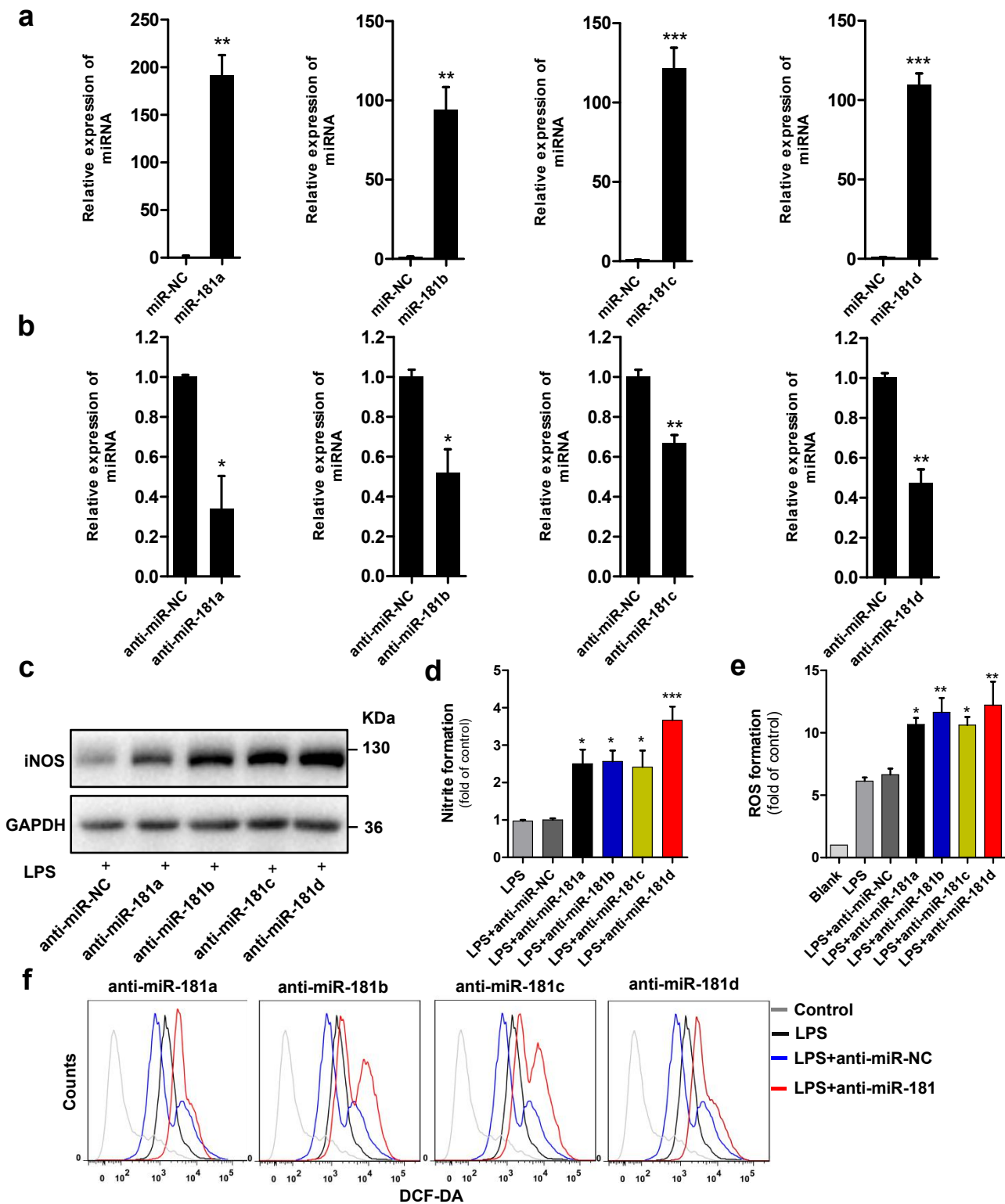

Supplement: Supplementary file 5 — supplementary figure 4 [file 41419_2018_821_MOESM5_ESM.pdf]

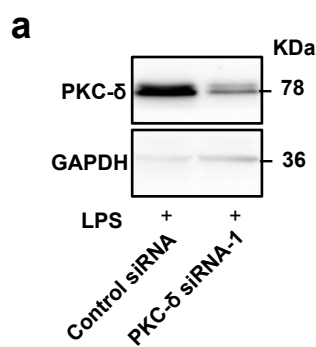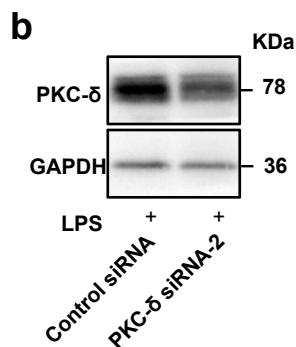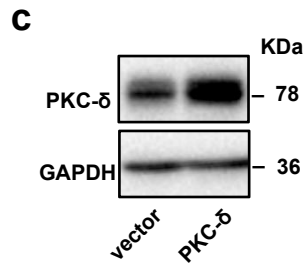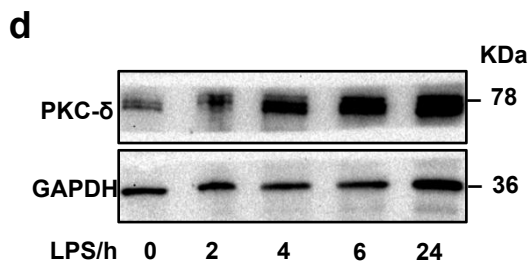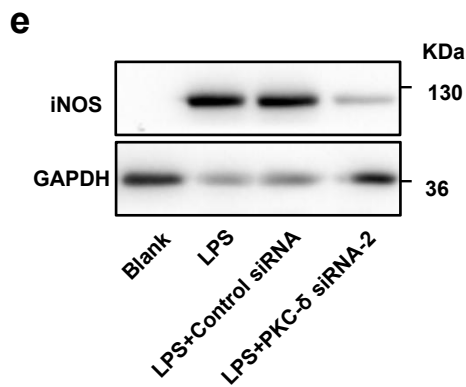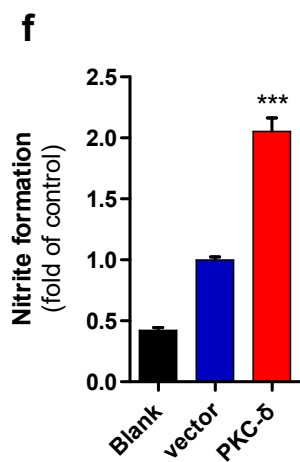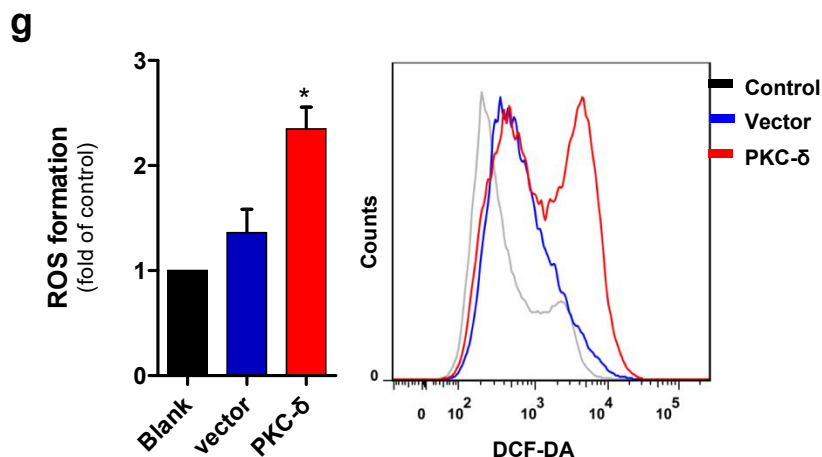

Supplement: Supplementary file 6 — supplementary figure 5 [file 41419_2018_821_MOESM6_ESM.pdf]

**a**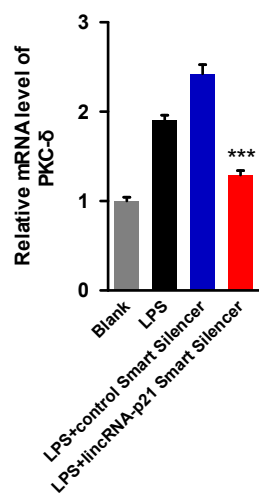**b**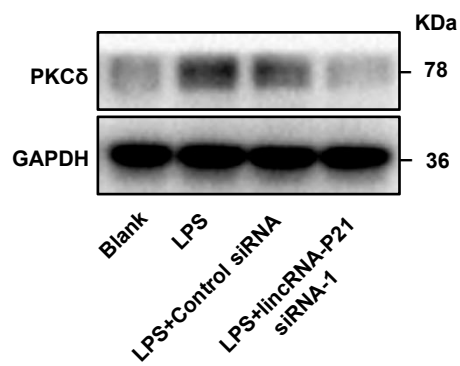**c**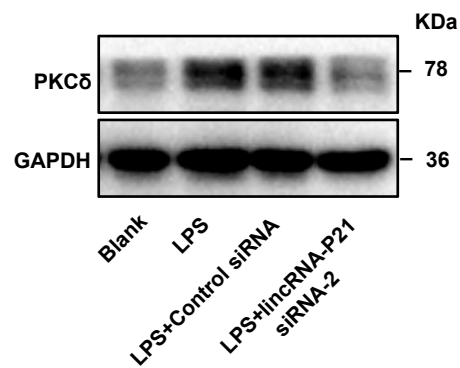**d**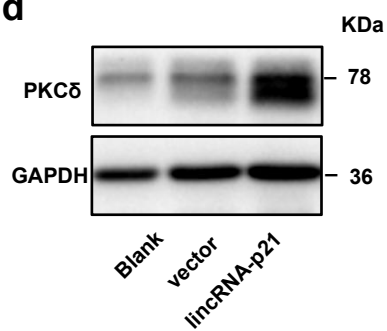**e**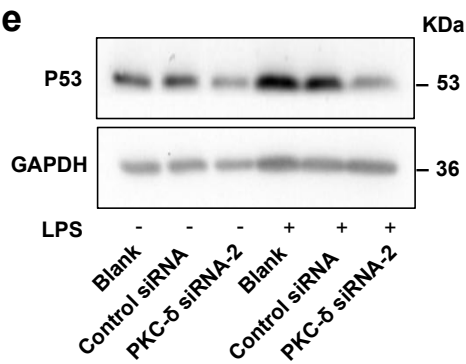

Supplement: Supplementary file 7 — supplementary figure 6 [file 41419_2018_821_MOESM7_ESM.pdf]

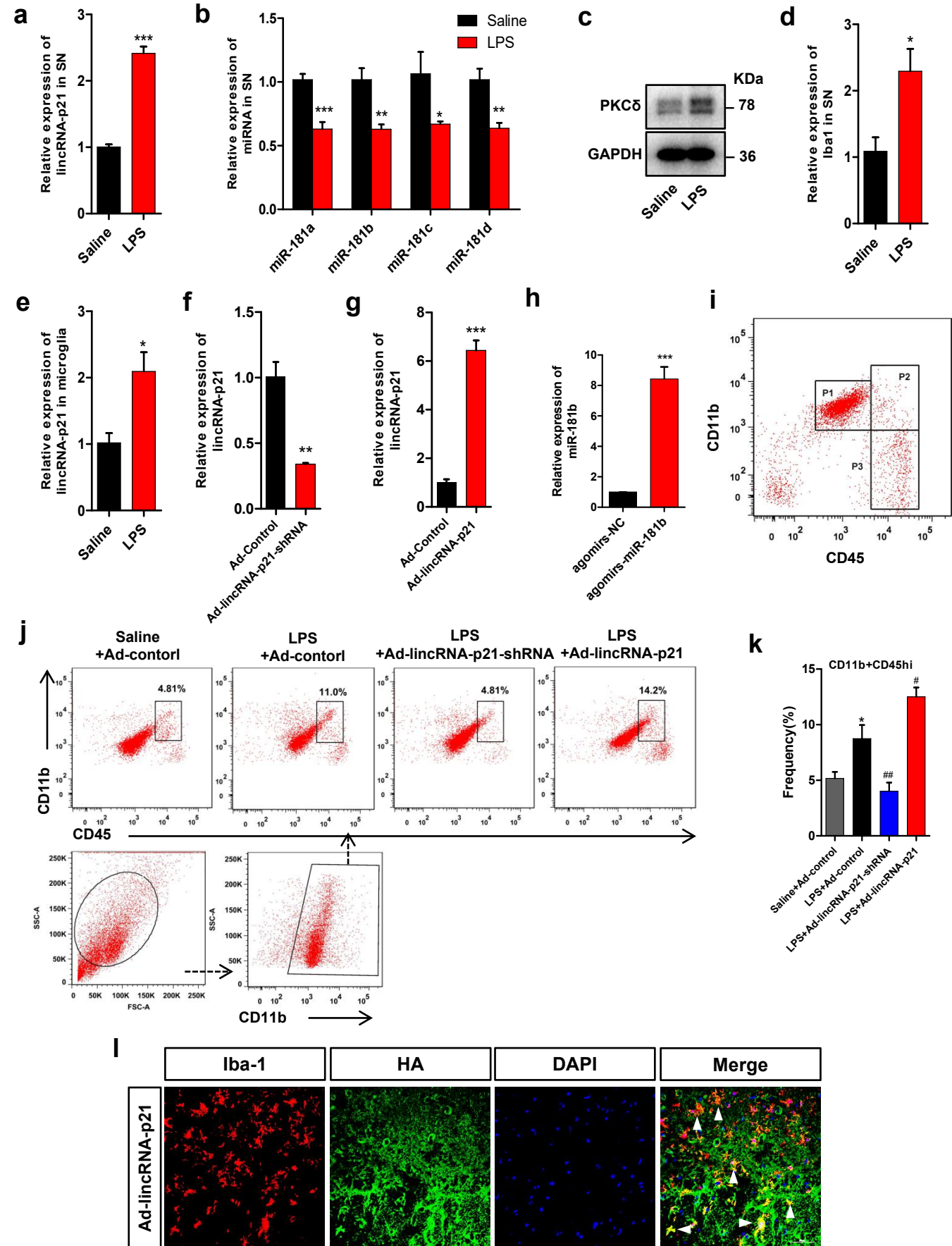

Supplement: Supplementary file 8 — supplementary figure 7 [file 41419_2018_821_MOESM8_ESM.pdf]

**a**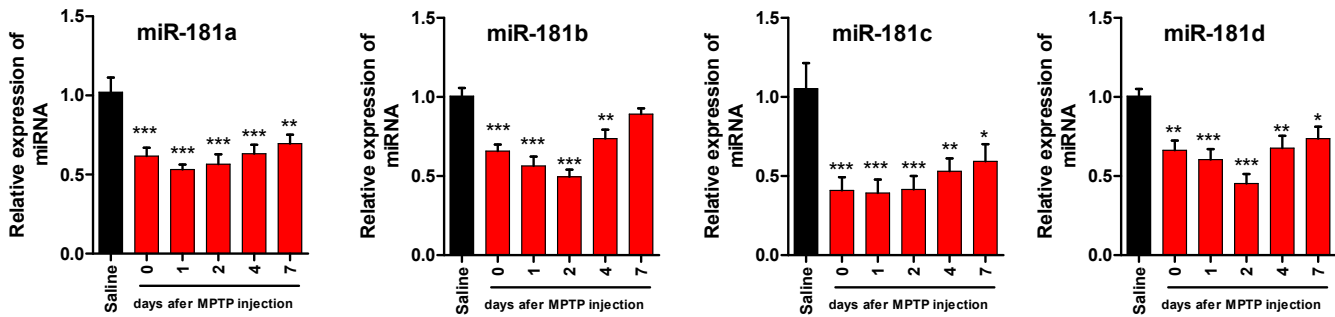**b**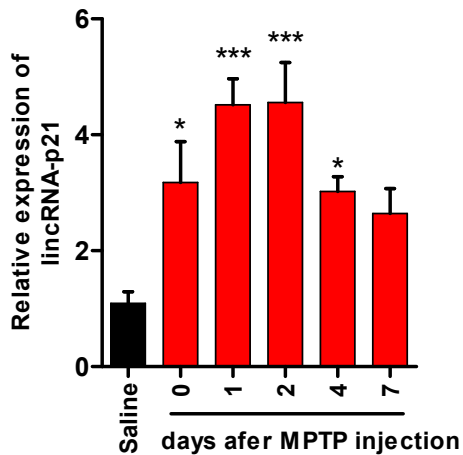**c**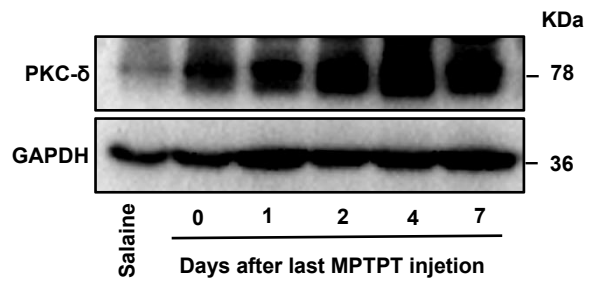**d**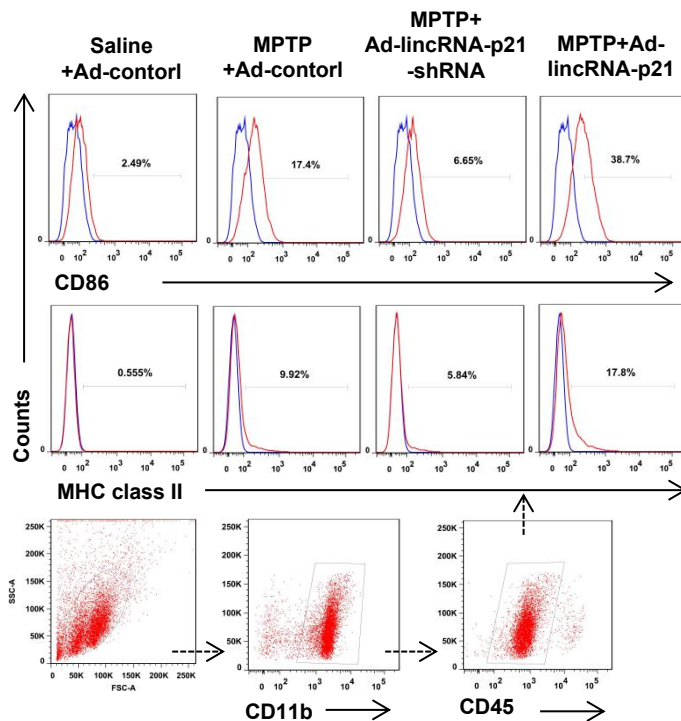**e**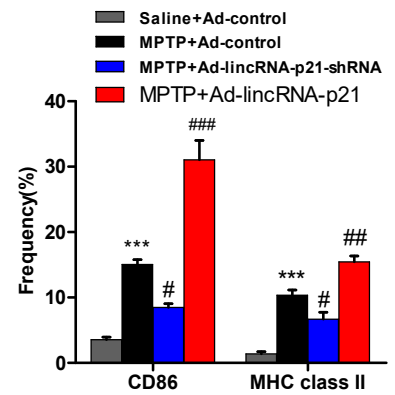

Supplement: Supplementary file 9 — supplementary figure 8 [file 41419_2018_821_MOESM9_ESM.pdf]
