# Supplementary material for: A lincRNA-p21/miR-181 family feedback loop regulates microglial activation during systemic LPS- and MPTP- induced neuroinflammation
Source: Cell Death Dis. 2018 Jul 23;9(8):803. doi: 10.1038/s41419-018-0821-5 (PMC6056543; doi:10.1038/s41419-018-0821-5)

Fig. 1c

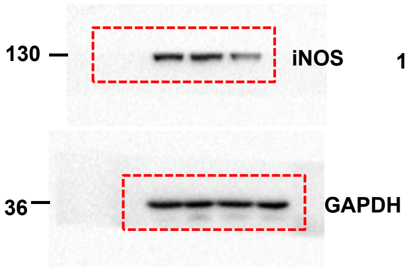

Fig. 1f

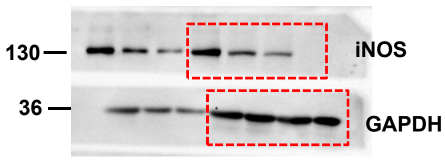

Fig. 2a

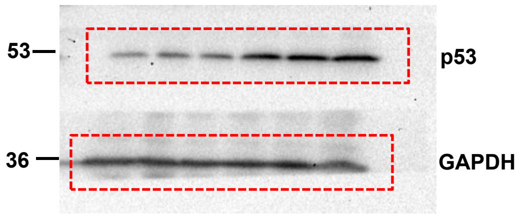

Fig. 2b

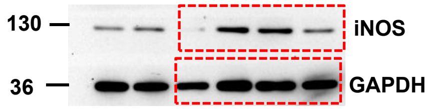

Fig. 2e

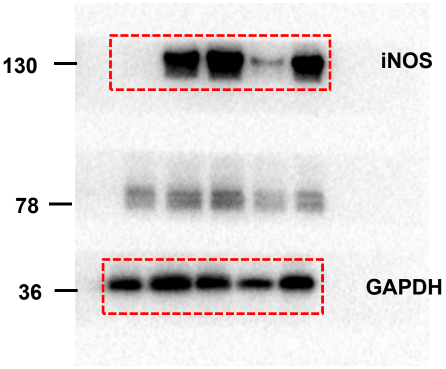

Fig. 3h

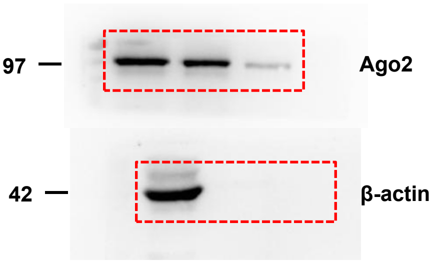

Fig. 4b

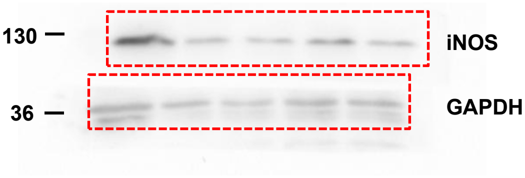

Fig. 4f

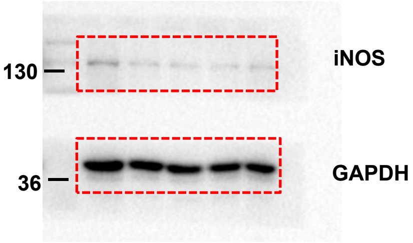

Fig. 5c

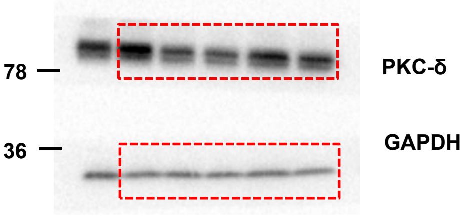

Fig. 5d

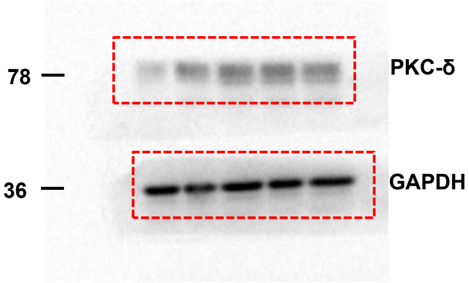

Fig. 5e

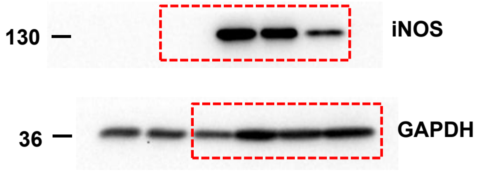

Fig. 6a

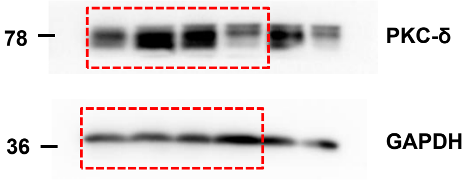

Fig. 6b

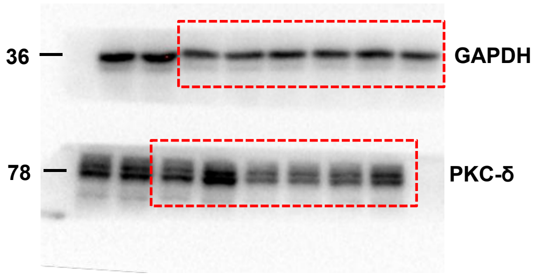

Fig. 6d

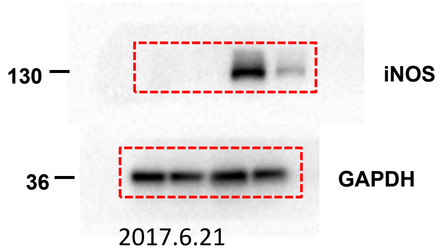

Fig. 6g

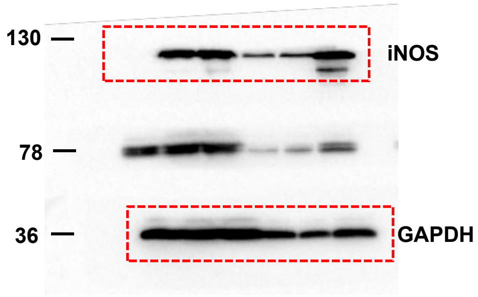

Fig. 6h

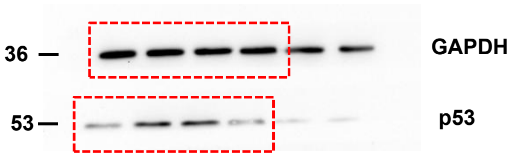

Fig. 6j

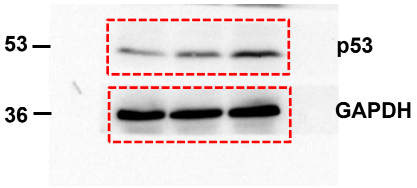

Fig. 6l

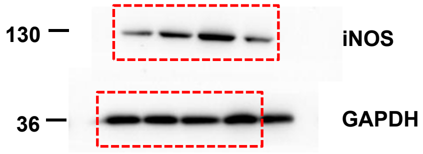

Fig. 6m

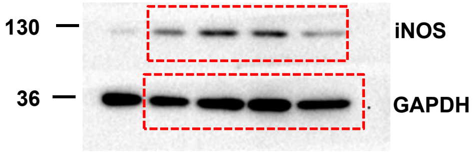

Fig. 7i

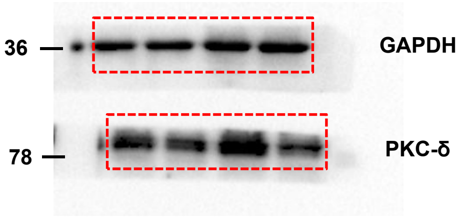

Fig. 7j

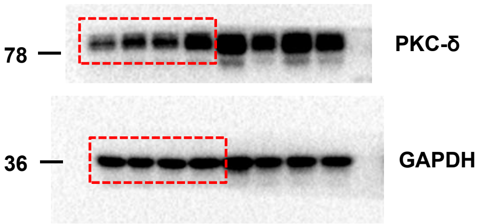

Supplement: Supplementary file 10 — supplementary figure 9 [file 41419_2018_821_MOESM10_ESM.pdf]
