# Supplementary material for: A lincRNA-p21/miR-181 family feedback loop regulates microglial activation during systemic LPS- and MPTP- induced neuroinflammation
Source: Cell Death Dis. 2018 Jul 23;9(8):803. doi: 10.1038/s41419-018-0821-5 (PMC6056543; doi:10.1038/s41419-018-0821-5)
Supplement: Supplementary file 11 — supplementary table 1 [file 41419_2018_821_MOESM11_ESM.pdf]

Supplementary Table 1

Gene		Primer sequences
lincRNA-p21	Forward	GAAAGCGAGTGGGACAGG
	Reverse	CAGGGCAAGAACTTGTGGAC
PRKCD	Forward	CCAAGGTGCTGATGTGTGTG
	Reverse	AAGGTGGCGATAAACTCGTG
iNOS	Forward	GCTTGGGTCTTGTTCACTCC
	Reverse	TCCTCTTTCAGGTCACTTTGG
IL-6	Forward	TTCCATCCAGTTGCCTTCTT
	Reverse	CATTTCCACGATTTCCCAGA
TNF-α	Forward	TATGGCTCAGGGTCCAACTC
	Reverse	GGAAAGCCCATTTGAGTCCT
IL-1β	Forward	TGTGCAAGTGTCTGAAGCAGC
	Reverse	TGGAAGCAGCCCTTCATCTT
MCP-1	Forward	CTGAAGCCAGCTCTCTCTTCCT
	Reverse	GAGCCAACACGTGGATGCT
Iba1	Forward	CTTGAAGCGAATGCTGGAGAA
	Reverse	GGAGCCACTGGACACCTCTCT
18S rRNA	Forward	CGGCTACCACATCCAAGGAA
	Reverse	GCTGGAATTACCGCGGCT
β-actin	Forward	CTAAGGCCAACCGTGAAAAG
	Reverse	ACCAGAGGCATACAGGGACA
U2 snRNA	Forward	GGCCTTTTGGCTAAGATCAAGT
	Reverse	GCAAGCTCCTATTCCAACTCC
miR-181a	Forward	GCGGCGGAACATTCAACGCTGTC
	RT primer	GTCGTATCCAGTGCAGGGTCCGAGGTA TTCGCACTGGATACGACACTCAC
miR-181b	Forward	TGG CGG AAC ATT CAT TGC TGT CG
	RT primer	GTCGTATCCAGTGCAGGGTCCGAGGTA TTCGCACTGGATACGACACCCAC
miR-181c	Forward	TTG GCG GAA CAT TCA ACC TGT CG
	RT primer	GTCGTATCCAGTGCAGGGTCCGAGGTA TTCGCACTGGATACGACACTCAC
miR-181d	Forward	CGG CGG AAC ATT CAT TGT TGT CG
	RT primer	GTCGTATCCAGTGCAGGGTCCGAGGTA TTCGCACTGGATACGACACCCAC
miRNA	Reverse	ATCCAGTGCAGGGTCCGAGG
